# MOSAIC: A Multiscale Model of Osteogenesis and Sprouting Angiogenesis with Lateral Inhibition of Endothelial Cells

**DOI:** 10.1371/journal.pcbi.1002724

**Published:** 2012-10-11

**Authors:** Aurélie Carlier, Liesbet Geris, Katie Bentley, Geert Carmeliet, Peter Carmeliet, Hans Van Oosterwyck

**Affiliations:** 1 Biomechanics Section, KU Leuven, Leuven, Belgium; 2 Prometheus, Division of Skeletal Tissue Engineering, KU Leuven, O&N 1, Leuven, Belgium; 3 Biomechanics Research Unit, University of Liege, Liege, Belgium; 4 Vascular Biology Lab, Cancer Research UK, London, United Kingdom; 5 Clinical and Experimental Endocrinology, KU Leuven, O&N 1, Leuven, Belgium; 6 Laboratory of Angiogenesis and Neurovascular link, Vesalius Research Center, VIB, Leuven, Belgium; 7 Laboratory of Angiogenesis and Neurovascular link, Vesalius Research Center, University of Leuven, Leuven, Belgium; Johns Hopkins University, United States of America

## Abstract

The healing of a fracture depends largely on the development of a new blood vessel network (angiogenesis) in the callus. During angiogenesis tip cells lead the developing sprout in response to extracellular signals, amongst which vascular endothelial growth factor (VEGF) is critical. In order to ensure a correct development of the vasculature, the balance between stalk and tip cell phenotypes must be tightly controlled, which is primarily achieved by the Dll4-Notch1 signaling pathway. This study presents a novel multiscale model of osteogenesis and sprouting angiogenesis, incorporating lateral inhibition of endothelial cells (further denoted MOSAIC model) through Dll4-Notch1 signaling, and applies it to fracture healing. The MOSAIC model correctly predicted the bone regeneration process and recapitulated many experimentally observed aspects of tip cell selection: the salt and pepper pattern seen for cell fates, an increased tip cell density due to the loss of Dll4 and an excessive number of tip cells in high VEGF environments. When VEGF concentration was even further increased, the MOSAIC model predicted the absence of a vascular network and fracture healing, thereby leading to a non-union, which is a direct consequence of the mutual inhibition of neighboring cells through Dll4-Notch1 signaling. This result was not retrieved for a more phenomenological model that only considers extracellular signals for tip cell migration, which illustrates the importance of implementing the actual signaling pathway rather than phenomenological rules. Finally, the MOSAIC model demonstrated the importance of a proper criterion for tip cell selection and the need for experimental data to further explore this. In conclusion, this study demonstrates that the MOSAIC model creates enhanced capabilities for investigating the influence of molecular mechanisms on angiogenesis and its relation to bone formation in a more mechanistic way and across different time and spatial scales.

## Author Summary

The healing of a fracture largely depends on the development of a new blood vessel network (angiogenesis), which can be investigated and simulated with mathematical models. The current mathematical models of angiogenesis during fracture healing do not, however, implement all relevant biological scales (e.g. a tissue, cellular and intracellular level) rigorously in a multiscale framework. This study established a novel multiscale platform of angiogenesis during fracture healing (called MOSAIC) which allowed us to investigate the interactions of several influential factors across the different biological scales. We focused on the biological process of tip cell selection, during which a specific cell of a blood vessel, the “tip cell”, is selected to migrate away from the original vessel and lead the new branch. After showing that the MOSAIC model is able to correctly predict the bone regeneration process as well as many experimentally observed aspects of tip cell selection, we have used the model to investigate the influence of stimulating signals on the development of the vasculature and the progression of healing. These results raised an important biological question concerning the criterion for tip cell selection. This study demonstrates the potential of multiscale modeling to contribute to the understanding of biological processes like angiogenesis.

## Introduction

### The process of angiogenesis during fracture healing

The biological process of fracture healing comprises three main stages: (i) the “inflammation phase”, where the trauma site becomes hypoxic and is invaded by inflammatory cells, fibroblasts, endothelial cells and mesenchymal stem cells [1]; (ii) the “reparative phase”, which starts with the production of cartilaginous and fibrous tissue resulting in a soft callus, later replaced by a hard callus, through the process of endochondral ossification; (iii) in the final “remodeling phase” the woven bone is replaced by lamellar bone and the vasculature is reorganized.

The healing of a fracture depends largely on the development of a new blood vessel network (angiogenesis) in the callus. Sprouting angiogenesis involves the following steps: first a “tip cell” is selected; this cell extends filopodia sensing the haptotactic and chemotactic cues in the environment and leads the newly formed “sprout” comprised of following, proliferating “stalk cells”; the newly formed sprout, or “branch” then connects with another branch in a process called anastomosis, which results in the formation of a closed loop allowing the initiation of blood flow; finally the newly formed vascular network is stabilized by pericytes [2].

In order to ensure a correct development of the vasculature, the balance between stalk and tip cell phenotypes must be tightly controlled. The process of tip cell selection consists of the following main steps. Firstly a gradient of vascular endothelial growth factor (VEGF) is formed by the up-regulation of VEGF-expression and secretion, triggered by hypoxia (low oxygen concentration). The VEGF-mediated activation of the VEGFR-2 receptors induces the up-regulation of Dll4 which activates the Notch1-receptors on the neighboring cells, thereby down-regulating their expression of VEGFR-2. This process of lateral inhibition, with cells battling to inhibit each other leads eventually to a “salt and pepper” alternating pattern, where cells with high Dll4 levels remain with high VEGFR-2 receptor levels, allowing them to migrate (and becoming tip cells) whilst their neighbors become inhibited, making them less susceptible to VEGF, and thus these adopt the non-migratory stalk cell phenotype. In this manner the adequate amount of tip cells, required for a correct sprouting pattern, is established [2]–[5].

### Multiscale models of angiogenesis

Both fracture healing as well as angiogenesis are very complex biological processes involving the coordinated action of many different cell types, biochemical and mechanical factors across multiple temporal and spatial scales. The time scales of individual events that underlie biological processes range from seconds for phosphorylation events to hours for mRNA transcription to weeks for tissue formation and remodeling processes [6]. The spatial scales vary from nanometers at the molecular level to millimeters at the tissue level and meters at the level of the organism [6], [7]. Thus, it can be concluded that most biological processes have an intrinsic multiscale nature and must be studied and modeled accordingly.

Depending on the biological spatial scale of interest a variety of experimental and modeling approaches can be used, which are nicely summarized by Meier-Schellersheim et al. [7]. The modeling approaches can be arranged in two broad categories: continuum and discrete modeling techniques. Continuum models use ordinary differential equations (ODEs) or partial differential equations (PDEs) to describe the evolution of cell and tissue densities and protein concentrations. The model variables are averages, which makes it difficult to represent individual cell-cell and cell-matrix interactions [8], [9]. Moreover, since the cells are not individually represented, it is challenging to model the individual intracellular processes. Also, continuum models fail to correctly capture the process of angiogenesis due to the inherent discreteness of vascular networks [10]. Discrete approaches are often used to study small-scale phenomena, e.g. biological processes at the cellular and subcellular levels [11]. However, these techniques often become computationally expensive when used for predictions of larger cell population sizes at the tissue scale [11].

Here we briefly review hybrid, multiscale models of angiogenesis, i.e. models that combine different modeling techniques for various scales mentioned above into one framework, as this is the approach we have adopted here. For comprehensive reviews on (multiscale) mathematical models of angiogenesis the reader is referred to Mantzaris et al., Qutub et al. and Peirce [12]–[14]. Notably none of the hybrid models to date include Dll4-Notch1 tip cell selection.

Milde et al. presented a deterministic hybrid model of sprouting angiogenesis where a continuum description of VEGF, MMPs, fibronectin and endothelial stalk cell density is combined with a discrete, agent-based particle representation for the tip cells [15]. The hybrid model describes the biological process of sprouting as the division of tip cells depending on chemo- and haptotactic cues in the environment and a phenomenological “sprout threshold age”. In the model of Lemon et al. the formation of a new branch also occurs at the tip cell position, but is modeled as a random process with an average number of branches per unit length of the capillary [16]. Checa et al. model sprout formation stochastically by making the probability of sprouting from a vessel segment proportional to the segment length. The capillary growth rate is also regulated by the local mechanical stimulus [17]. Peiffer et al. proposed a hybrid bioregulatory model of angiogenesis during bone fracture healing [18], based on the deterministic hybrid model of Sun et al. [19]. The process of angiogenesis is modeled discretely, including sprouting and anastomosis. The selection of tip cells in the growing vascular network is, however, modeled with phenomenological rules. A three dimensional model of cellular sprouting at the onset of angiogenesis was developed by Qutub et al. [20]. Although this framework describes sprout formation as a function of the local VEGF concentration and the presence of Dll4, it is only a first approximation of the complex tip-to-stalk cell communication by Dll4/Notch signaling.

A more detailed model of the lateral inhibition that underlies the tip cell selection process in angiogenic sprout initialization was presented by Bentley et al. [3], [21]. They use an agent-based framework to accurately simulate a small capillary comprising 10 endothelial cells that can change shape and sense the local VEGF concentration by extending very thin filopodia. Moreover, every endothelial cell is characterized by its individual protein levels of VEGFR-2, Dll4 and Notch – and their distribution on the cell membrane, by further subdividing the membrane into separate agents – which does not only allow assessment of the effects of the VEGF environment on tip/stalk cell patterning but also those of Dll4 over- and under-expression and cell shape change.

Sprouting angiogenesis involves multiple biological scales: the intracellular scale where gene expression is altered so that different phenotypes (e.g. tip and stalk cells) can arise, the cellular scale that involves proliferation and migration and the tissue scale that encompasses the concentration fields of soluble and insoluble biochemical factors. As these scales are highly coupled, multiscale models are needed to study the mechanisms of sprouting angiogenesis. To the best of the authors' knowledge, there is only one model of sprouting angiogenesis with Dll4-Notch1 rigorously implemented [3] but this model simplified the extracellular environment to a uniform or linearly varying field of VEGF concentration, which is constant in time. While this simplification is justified for a detailed study of the short term phenotypic changes of a few neighboring endothelial cells, it is not for more complex, multicellular systems that involve cell-matrix interaction and highly dynamic, extracellular environments. This is certainly true for fracture healing, in which matrix densities and (gradients of) extracellular concentrations of soluble signals, like VEGF, are spatially and temporally changing as a result of cellular activity. While efforts have been done to model the interplay of VEGF diffusion and sprouting angiogenesis in the context of skeletal muscle tissue [6], these multiscale models did not incorporate Dll4-Notch1 signaling. Moreover, in the context of fracture repair multiscale models that consider angiogenesis and that relate tissue, cell and intracellular scales have not been established yet [22].

### Objectives of this study

In this study, we present a multiscale model of osteogenesis and sprouting angiogenesis with lateral inhibition of endothelial cells (MOSAIC) which extends the bioregulatory framework of Peiffer et al. [18] with an intracellular model based on the work of Bentley et al. on tip cell selection [3]. We hypothesize that the MOSAIC model creates enhanced capabilities for investigating the influence of molecular mechanisms on angiogenesis and its relation to bone formation. Simulation results will illustrate the interplay between molecular signals, in particular VEGF, Dll4 and Notch1, endothelial cell phenotypic behavior and bone formation. They will demonstrate the advantages of multiscale modeling in the context of fracture healing, thereby exploring the importance of the model of Bentley et al. [3] for a much more complex and dynamic extracellular environment. At the same time, by comparison to the more phenomenological model of Peiffer et al. [18] the potential of a more mechanistic treatment of tip cell selection will become clear.

## Methods

**Figure 1:**
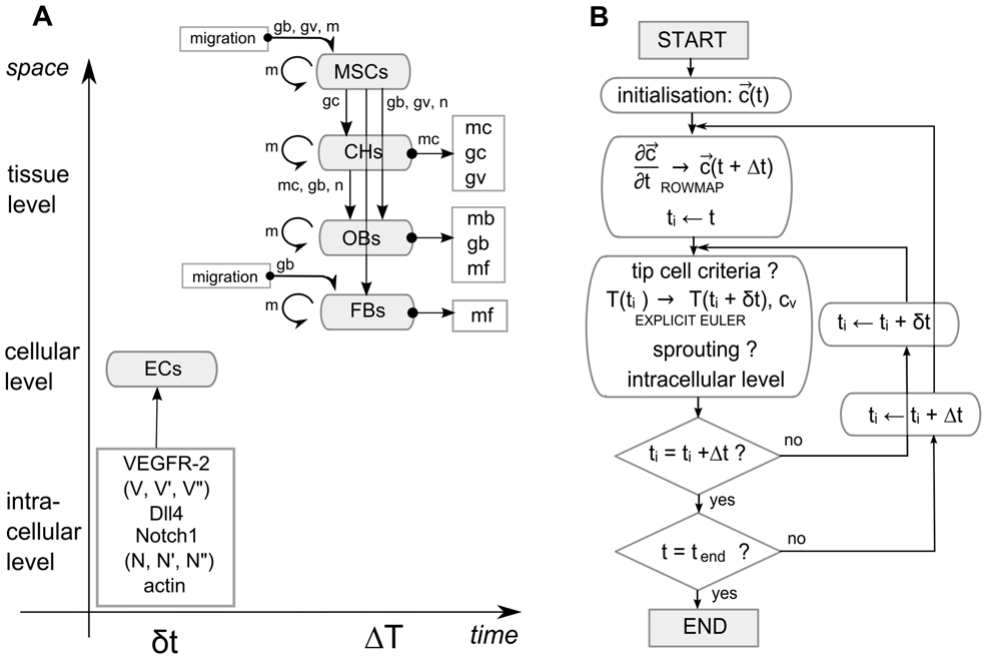
Schematic overview of the multiscale model and a flowchart of its numerical implementation. (A) Scale separation map indicating schematically the modeled processes at different spatial and temporal scales. Intracellular variables are further explained in Table 1 and govern endothelial cell (EC) behavior. Cell types that are considered at the tissue scale (MSCs: mesenchymal stem cells, CHs: chondrocytes, OBs: osteoblasts, FBs: fibroblasts) can migrate (only MSCs and FBs), proliferate (circular arrows), differentiate (vertical arrows) and produce growth factors (*gb*: osteogenic growth factor concentration, *gc*: chondrogenic growth factor concentration, *gv*: angiogenic growth factor concentration) and extracellular matrix (*mf*: fibrous tissue density, *mb*: bone density, *mc*: cartilage density, *m*: total tissue density). Blood vessels serve as an oxygen source (*n*: oxygen concentration). Variables next to an arrow indicate their mediating role for a certain tissue level process. (B) Flowchart of the numerical implementation of the MOSAIC model (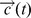: vector of the continuous variables, *t*: time, *δt*: time step of the inner loop, *Δt*: time step of the outer loop, *c_v_*: endothelial cells).

The MOSAIC model presented in this work integrates an intracellular module based on the work of Bentley et al. [3] into the model of Peiffer et al. [18]. Figure 1 gives a schematic overview of the MOSAIC model which consists of (1) a tissue level describing the various key processes of bone regeneration with continuous variables, (2) a cellular level representing the developing vasculature with discrete endothelial cells and (3) the intracellular level that defines the internal dynamics of every endothelial cell. The combination of continuous and discrete modeling techniques results in a hybrid, multiscale model.

### Discrete description of angiogenesis

The discrete variable *c_v_* represents the blood vessel network, which is implemented on a lattice. When a grid volume contains an endothelial cell this variable is set to 1, otherwise *c_v_* = 0. The vessel diameter is defined by the grid resolution and is always one endothelial cell wide, although the movement of the tip cell is grid independent as explained below. Every endothelial cell (*c_v_* = 1) has unique intracellular protein levels, which control the behavior of that specific cell. The intracellular module is adapted from the agent-based model of Bentley et al. [3] and consists of the following variables: the level of VEGFR-2 (*V*), Notch1 (*N*), Dll4 (*D*), active VEGFR-2 (*V′*), active Notch1 (*N′*), effective active VEGFR-2 (*V″*), effective active Notch1 (*N″*) and the amount of actin (*A*). The effective active levels (*V″* and *N″*) include the time delay of translocation to the nucleus, thereby representing the levels at the nucleus, influencing gene expression. The amount of actin (*A*) refers to the polymerized actin levels (F-actin) inside the cell. In particular, it is associated to actin used for filopodia formation, owing to its importance for tip cell migration. As such, an increase in actin levels can be interpreted as filopodia extension, while a decrease as filopodia retraction. Other intracellular signaling pathways that involve actin, such as energy metabolism [23], [24], are not considered.

The following equations describe the intracellular dynamics. An overview of all the parameters of the intracellular module can be found in Table 1.

**Table 1:** Parameter values of EC state variables.

Parameter	Definition	Setting
*M_tot_*	number of membrane agents per EC	1500
*V_max_*	maximal amount of VEGFR-2 receptors	115000
*V_min_*	minimal amount of VEGFR-2 receptors	2500
*N*	amount of Notch receptors (constant)	25000
*D_max_*	maximal amount of Dll4	25000
*A_max_*	maximal amount of actin	5000
*V_sink_*	proportion of VEGF left by VEGFR-1	0.275
*σ*	VEGFR-2 expression change due to Notch1	47.40
V′*	threshold of active VEGFR-2 for actin production and migration of tip cells	200
A*	threshold of actin for tip cell selection	3500
V*	threshold of VEGFR-2 for tip cell selection	*V_max_*/2
*ΔA*	constant increment of actin	50
*D_1_*	delay between active and effective active VEGFR-2 levels	3.δt
*D_2_*	delay between active and effective active Notch levels	3.δt
*D_3_*	inactive time before retraction of filopodia	3.δt
*ee*	maximal time step of inner loop	1.2 h
*row*	maximal time step of outer loop	8.57 h

**Figure 2:**
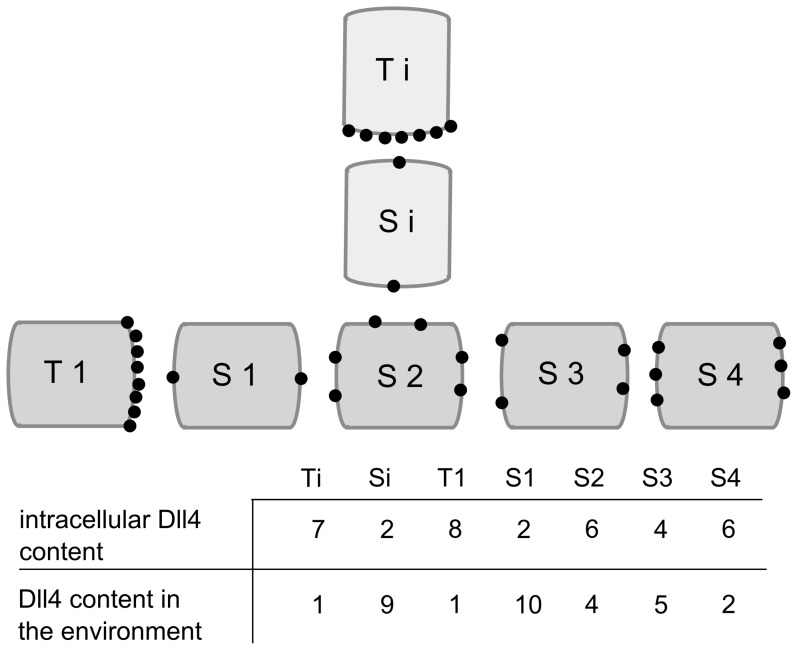
Conceptual representation of the Dll4 distribution across the cell membranes of endothelial cells. The dark grey ECs represent the original branch whereas Ti and Si are part of a newly formed sprout (T = tip cell, S = stalk cell). The amount of Dll4 was arbitrarily chosen, a maximal amount of 25 000 ligands per EC (for an EC with a length of 25 µm) was calculated by Bentley et al. [3], estimated from Liu et al. [63].

The activation of the VEGFR-2 receptor, described by *V′*, is given by: 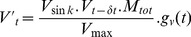
(1)where the constant *V_sink_* represents the amount of VEGFR-1 receptors that act as a sink (decoy receptor) by removing VEGF from the system, *t* represents the time and *δt* the time step of the inner loop (more information on these parameters can be found in the section “implementation details” below), *V_max_* represents the maximal amount of VEGFR-2 receptors, *g_v_* is the local VEGF concentration (at the tissue level) and *M_tot_* is the total number of membrane agents (constant for all ECs). Equation 1 is adapted from Bentley et al. [3] where every EC is composed of a varying amount of membrane agents, representing small sections of the cellular membrane. In the current framework, however, every EC is represented by one agent so that *M_tot_* was chosen to be constant for all ECs and equal to an intermediate value between the initial and maximal amount of membrane agents in the agent-based framework of Bentley et al. [3]. The level of activated VEGFR-2 remains in a range going from 0 to *V*. When the VEGFR-2 receptors are activated above a certain threshold (V′*), the actin levels of the endothelial cell are incremented in a constant manner (*ΔA*). As mentioned earlier, this represents the extension of filopodia by the endothelial cell, which is shown to be regulated downstream of VEGFR-2 [4]. If the endothelial cell fails to extend its filopodia for a certain amount of time *D_3_*, the filopodia retract which is mathematically translated into a reduction of the actin levels in a constant manner (−10.*ΔA*). The actin level remains in a range between 0 and *A_max_*. The amount of Notch1 is considered to be constant in every EC, representing a balance between the rate of degradation and expression. At the same time, initial Notch activity levels are assumed to be zero and in the model Notch activity can only be increased through binding with Dll4 from neighboring ECs. The model therefore neglects the role of Notch in EC quiescence and the fact that high Notch activity levels have been measured in quiescent ECs [25]–[27]. Instead, it only focuses on the role of Dll4-Notch in tip cell selection. The number of activated Notch receptors (N′) will be equal to the total amount of Dll4 available (with an upper bound, given by the total number of Notch receptors N). The amount of Dll4 in the environment of an EC is the sum of the amount of Dll4 at the junctions with its neighboring ECs, whereby every cell is assumed to distribute Dll4 uniformly across its cell-cell junctions (see Figure 2).

After a delay of *D_1_* for *V′* and *D_2_* for *N′* the active VEGFR-2 and Notch levels become the effective active levels (*V″* and *N″*) representing the levels at the nucleus, influencing gene expression. The delays were taken from Bentley et al. which were fitted to a somite clock Delta-Notch system [3], [28]. Note that there is a delay between receptor activation and gene expression (transcription) but not between gene expression and protein synthesis (translation), which is a simplification of the model.

The amount of Dll4 is modeled in the following way:
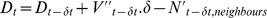
(2)

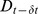
 represents the previous amount of Dll4, 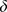 the change in Dll4 expression due to the activation of the VEGFR-2 receptor [4], [29] and 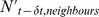 is the amount of Dll4 that is removed from the environment due to the activation of Notch-receptors on neighboring ECs. If-conditions are used to ensure that the Dll4 level remains in a range between 0 and *D_max_*. When Notch signaling is activated in a cell, the amount of VEGFR-2 receptors is down-regulated, suppressing the tip cell phenotype as follows [4], [5]:
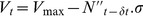
(3)
*V_max_* represents the maximal amount of VEGFR-2 receptors and 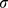 models the VEGFR-2 expression change due to Notch1 activation. If-conditions are used to ensure that the VEGFR-2 level remains in a range going from *V_min_* to *V_max_*. Since the amount of VEGFR-2 (*V*) at the previous timestep (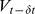) is not present in Equation 3, the amount of VEGFR-2 is continuously in equilibrium with the amount of effective active Notch1 (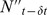). Equation 3 implies that in quiescent cells the number of VEGFR-2 receptors will be maximal, owing to the absence of any Notch activity. As mentioned earlier, the model neglects the role of Notch activity in quiescence and the fact that it will lead to reduced VEGFR-2 levels in quiescent ECs [25]–[27].

Note that Bentley et al. [3] represent every EC by a varying number of agents (to account for changes in cell shape and cell growth), whereas in this study every EC is represented by one agent. However, in order to use the parameter values and equations (in an adapted form) of Bentley et al. [3], *M_tot_* was fixed at a constant value for all ECs. Consequently, the values of *V*, *N*, *V′*, *N′*, *V″*, *N″*, *D* and *A* are evaluated at the cellular level, not at the level of individual membrane agents. This also implies that here cellular polarity is not captured explicitly as receptor and ligand concentrations are uniformly distributed across the membrane junctions. In the current model cell directional behavior follows from gradients of extracellular signals alone.

The evolution of the vascular network is determined by tip cell movement, sprouting and anastomosis [18], [19], outlined below.

#### Tip cell movement

The model computes the movement of every tip cell in a lattice-free manner. The cells that trail behind this tip are subsequently considered to be endothelial cells. Consequently, although the movement of a tip cell is grid independent, the vessel diameter is defined by the grid resolution due to the projection of the blood vessels on the grid. The movement of the tip cells is determined by their direction and speed, which is described by the tip cell velocity equations:
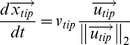
(4)where 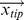 represents the position, 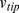 the speed and 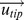 the direction of movement of the tip cell. The tip cell speed depends on the active VEGFR-2 concentration, meaning that both the surrounding VEGF concentration as well as the amount of VEGF-receptors influences the behavior of the tip cell [4], [30]. Below a threshold activation level (V′*) the tip cells do not migrate, above this, the tip cell velocity increases with *V′* up to a maximal speed of 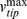. This translates into the following equation, where a third order polynomial was used to ensure a smooth threshold [18]:
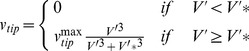
(5)The direction of movement is influenced by chemotactic and haptotactic signals and is modeled in the same way as Peiffer et al. [18].

#### Sprouting

The tip cell phenotype is induced (formation of a new branch) or maintained (in already existing tip cells) if the following requirements are satisfied:
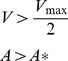
(6)This criterion means that the endothelial cell must have enough VEGFR-2 and filopodia (polymerized actin) to sense the environment and direct the trailing branch towards the source of VEGF.

#### Anastomosis

When a tip cell encounters another blood vessel anastomosis takes place, after which the EC loses its tip cell phenotype.

### Evolution of the continuous variables

At the tissue level, the fracture healing process is described as a spatiotemporal variation of eleven continuous variables: mesenchymal stem cell density (*c_m_*), fibroblast density (*c_f_*), chondrocyte density (*c_c_*), osteoblast density (*c_b_*), fibrous extracellular matrix density (*m_f_*), cartilaginous matrix density (*m_c_*), bone extracellular matrix density (*m_b_*), generic osteogenic growth factor concentration (*g_b_*), chondrogenic growth factor concentration (*g_c_*), vascular growth factor concentration (*g_v_*) and concentration of oxygen (*n*). The set of partial differential equations (PDEs) accounts for various key processes of bone regeneration. Initially the callus is filled with granulation tissue and the mesenchymal stem cells and growth factors will quickly occupy the regeneration zone. Subsequently the mesenchymal stem cells differentiate into osteoblasts (intramembranous ossification – close to the cortex away from the fracture site) and chondrocytes (central callus region). This is followed by VEGF expression by (hypertrophic) chondrocytes, which attracts blood vessels and osteoblasts and which is accompanied by cartilage degradation and bone formation (endochondral ossification). The model does not include bone remodeling. The general structure of the set of continuous equations is given by: 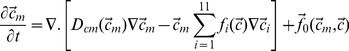
(7)

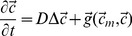
(8)where *t* represents time, 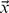 the space and 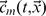 the density of a migrating cell type (mesenchymal stem cells and fibroblasts). 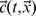 represents the vector of the other nine concentrations, ECM densities, growth factor concentrations and oxygen concentrations for which no directed migration is modeled. 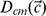 and 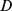 are the diffusion coefficients. 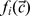 represents the taxis coefficients for both chemotaxis and haptotaxis. 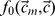 and 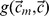 are reaction terms describing cell differentiation, proliferation and decay and matrix and growth factor production and decay. Detailed information, including the complete set of equations, boundary and initial conditions, parameter values and implementation details can be found in Peiffer et al. [18] and Geris et al. [31] and are provided here as online supplement.

### Implementation details

The partial differential equations are solved on a 2D grid with a grid cell size of 25 µm. The width of the discrete ECs is determined by the size of a grid cell (25 µm). Since the ECs in the model of Bentley et al. [3] have a width of 10 µm, the parameter values taken from Bentley et al. are multiplied with a factor of 2.5 (see Table 1). The partial differential equations are spatially discretized using a finite volume method assuring the mass conservation and nonnegativity of the continous variables [32]. The resulting ODEs are solved using ROWMAP, a ROW-code of order 4 with Krylov techniques for large stiff ODEs [33]. The MOSAIC model is deterministic and implemented in Matlab (The MathWorks, Natick, MA).

The flowchart in Figure 1B gives a schematic overview of the computational algorithm used in this study. Firstly the continuous variables are calculated. Then the inner loop is iterated which consists of four subroutines: (1) the current tip cells are evaluated by the tip cell selection criterion and, if necessary, they lose their tip cell phenotype; (2) the new position of every tip cell is calculated using a central difference scheme in space in combination with explicit Euler time integration; (3) the code checks whether sprouting occurs, meaning that other endothelial cells also satisfy the criterion for tip cell selection; (4) the intracellular levels of every endothelial cell are updated. Finally, the inner and outer loops are iterated until the end time of the simulation is reached.

The outer loop has a maximal time step size of 8.57 hours (*row*). Since the tip cells do not move more than one grid cell (25 µm) in this time interval (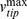 = 35 µm/day [19]), this maximal time step size (*row*) implies that the 11 PDEs can be solved for a constant vasculature. The inner loop has a maximal step size of 1.2 hours (*ee*), similar to Peiffer et al. [18], and was chosen so that the movement of the tip cells within one grid cell could be accurately followed (*ee*≪*row*). To reduce implementation difficulties, the time step of the inner loop (*δt*) is determined by calculating how many maximal inner loop time steps (*ee*) can fit in one outer loop time step (*ΔT*) and dividing the outer loop time step by this number. Consequently, the time step of the inner loop is not constant, which means that *D_1_*, *D_2_* and *D_3_* vary slightly, but this is a minor trade-off for the computational efficiency. Numerical convergence tests have shown that the average inner time step *δt* is equal to 155 s. Consequently, *D_1_*, *D_2_*, *D_3_* approximate the delays chosen by Bentley et al. [3]. Since the time step *δt* is approximately 10 times the time step of Bentley et al. [3], the parameter values of *σ* and *δ* have been altered to match the dynamics of the Dll4-Notch system. Numerical tests have shown that similar behavior is retrieved when both *σ* and *δ* are multiplied with 3.16 (see Table 1).

### Simulation details

**Figure 3:**
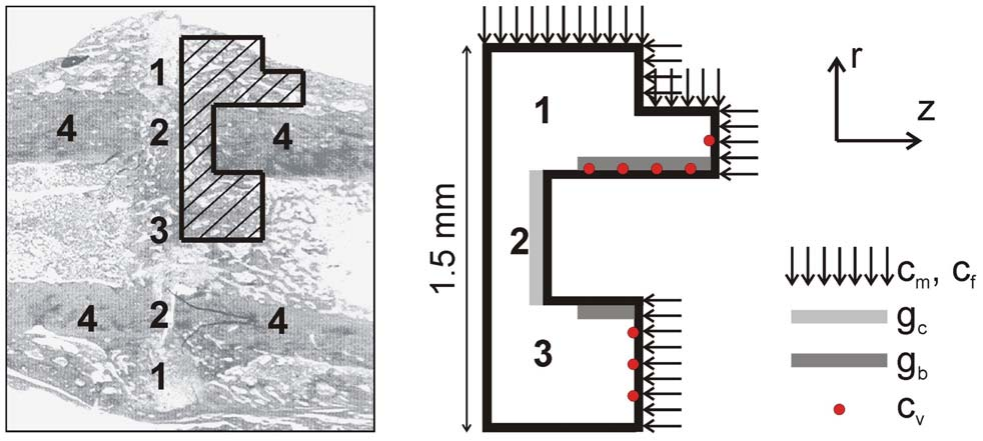
Geometrical domain and boundary conditions. (left) Geometrical domain deduced from the real callus geometry at postfracture week 3 [35]; 1 periosteal callus; 2 intercortical callus; 3 endosteal callus; 4 cortical bone. (right) Boundary conditions. The mesenchymal stem cells (*c_m_*) and fibroblasts (*c_f_*) are released from the periosteum, the surrounding soft tissues and the bone marrow [64]. The chondrogenic growth factors (*g_c_*) are released from the degrading bone ends [65] whereas the cortex is modeled to be the source of osteogenic growth factors (*g_b_*) [66]. *c_v_* indicates the initial position of the ECs.

Simulations were conducted using a quad-core Intel® Xeon® CPU with 12 GB RAM memory. Initially the callus domain is filled with granulation tissue only (*m_f,init_* = 10 mg/ml), all other continuous variables are initialized to zero. Boundary conditions are presented in Figure 3. Further information on the choice of appropriate boundary and initial conditions of the continuous variables can be found in Peiffer et al. [18] and Geris et al. [31].

#### Normal fracture healing

The initial values of the endothelial cell variables are summarized in Table 2. The EC starting positions are depicted in Figure 3.

**Table 2:** Initial values of EC state variables.

Variable	Definition	Setting
*V*	level of VEGFR-2	*V_max_*
*N*	level of Notch1	N
*D*	level of Dll4	0
*V′*	level of active VEGFR-2	0
*N′*	level of active Notch1	0
*V″*	level of effective active VEGFR-2	0
*N″*	level of effective active Notch1	0
*A*	level of actin	5000

#### Impaired angiogenesis

The influence of very high VEGF concentrations on tip/stalk cell patterning and angiogenesis was investigated by including an additional, constant source term (constant production rate) in the equation for *g_v_*. This term was varied between 0% and 10% of the default production rate (*G_gvc_* since the chondrocytes dominate the angiogenic growth factor production; see supplementary material for the explanation of the meaning of *G_gvc_*) representing normal and very high VEGF concentrations in the callus respectively. The effect of the injection of VEGF-antibodies, described by an additional sink term in the equation for *g_v_*, was also simulated [34]. The sink term was defined in such a way that no more VEGF than present could be removed thereby ensuring that the VEGF concentration stays positive. The effect of pharmacological inhibition of the VEGFR-2 receptor was investigated by setting *V′* to zero.

#### Sensitivity analysis

As Peiffer et al. [18] already performed extensive sensitivity analyses additional sensitivity analyses focused on the parameters related to the newly implemented tip cell selection mechanism (i.e. *σ*, *δ*, V′*, *V_sink_*). Experimental observations indicate that heterozygous Dll4 knockout mice still have some Notch activity but produce too many tip cells due to the lowered inhibition levels [5]. The MOSAIC model was used to simulate various genotypes corresponding to different expression levels of Dll4. The parameter *δ*, which defines the Dll4 expression change due to VEGFR-2 activation, was varied between 50% and 200%, representing a heterozygous knockout genotype and overexpression respectively [3]. The parameter *σ*, which represents the influence of the VEGFR-2 expression change due to Notch1, was also varied, between 33% and 133%. The threshold of active VEGFR-2 for actin production and migration of tip cells (V′*) was varied between 25% and 1000%. The amount of VEGFR-1 acting as a decoy receptor was changed by reducing *V_sink_* from 0.9% to 364%. The effect of the initial values of Dll4 (*D_0_*) and actin (*A_0_*) was also investigated, the initial amount of Dll4 was varied between *D_min_* and *D_max_* and the initial amount of actin was varied between 0% and 100%.

## Results

### Normal fracture healing

**Figure 4:**
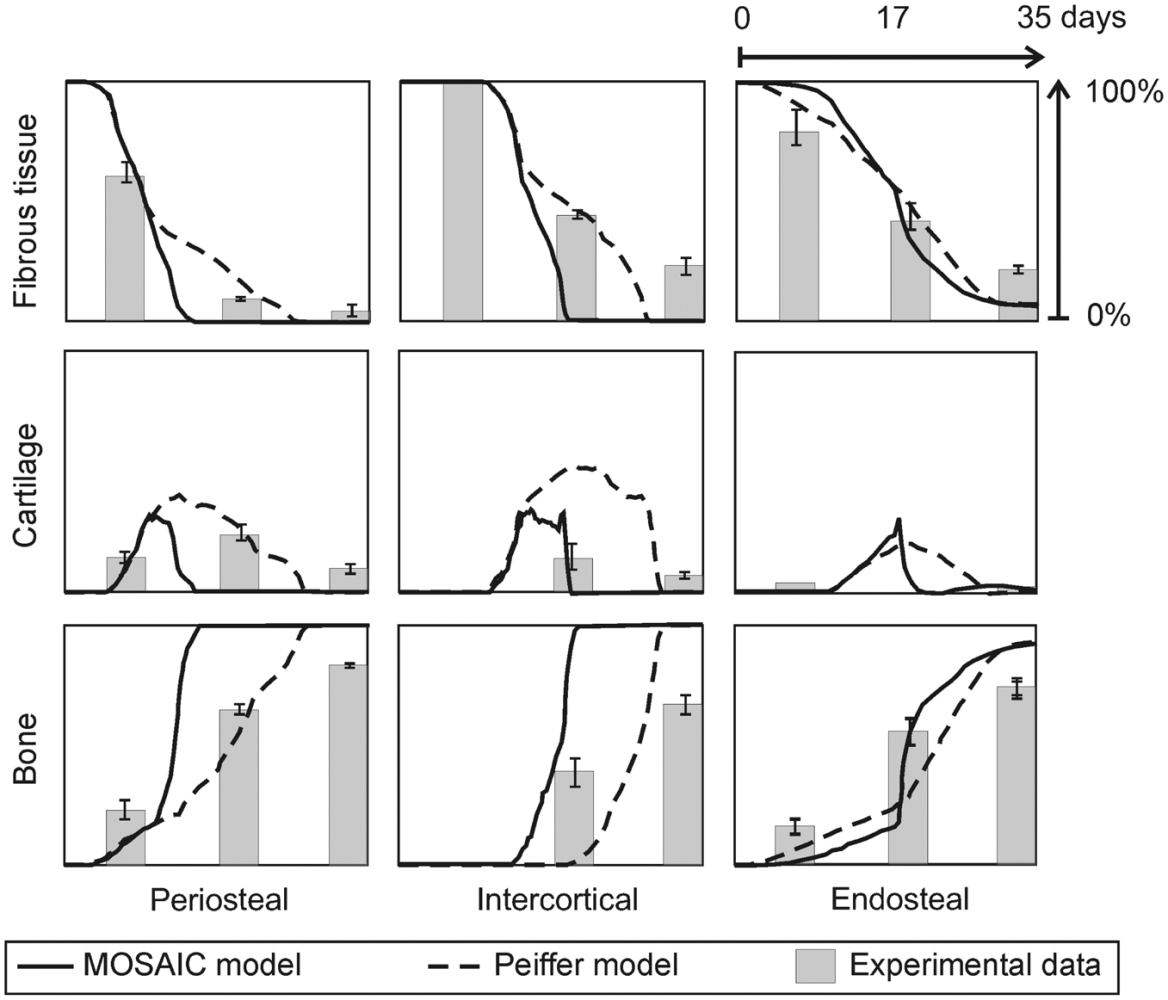
*In silico and in vivo* evolution of normal fracture healing. Temporal evolution of the bone, cartilage and fibrous tissue fractions (%) in the periosteal, intercortical and endosteal callus as predicted using the hybrid model of Peiffer et al. [18] and the newly developed multiscale model and as measured by Harrison et al. [35].

The MOSAIC model predicts the evolution of the continuous variables as well as the evolution of the intracellular variables during normal fracture healing. The osteoprogenitor cells enter the callus from the surrounding tissues and differentiate into osteoblasts under the influence of osteogenic growth factors. This leads to rapid intramembranous ossification near the cortex and distant from the fracture line. In the endosteal and intercortical callus the bone is formed through the endochondral pathway, starting from a cartilage template that is mineralized as the blood vessel network is formed to supply the complete fracture zone with oxygen. Figure 4 compares the predictions of the Peiffer-model [18] and the MOSAIC model with the experimentally measured tissue fractions of Harrison et al. in a rodent standardized fracture model [35]. Both models capture the general trends in the experimental data equally well: the bone tissue fraction gradually increases throughout the healing process; the fibrous tissue fraction disappears; the cartilage template is first produced and later replaced by bone.

After one, two and three weeks of simulated healing time the surface fraction of the blood vessels in the callus is respectively 2.34%, 18.20% and 46.25%. Experimental results also show that the vascular plexus is very dense in the fracture callus, although quantitative results are lacking [36], [37]. Images, illustrating the angiogenic and osteogenic process in the fracture callus can be found in Maes et al. and Lu et al. [38], [39]. These experimental studies report that at the progressing front, there is a tree-like structure of tip cells extending filopodia to sense their environment and to guide the developing sprout. At the back, the vasculature is being remodeled into a more structured network of larger vessels with more quiescent endothelial cells. At present this remodeling phase of the vasculature, which will remove some blunt ends as well as redundant vessels, is not included in the MOSAIC model.

**Figure 5:**
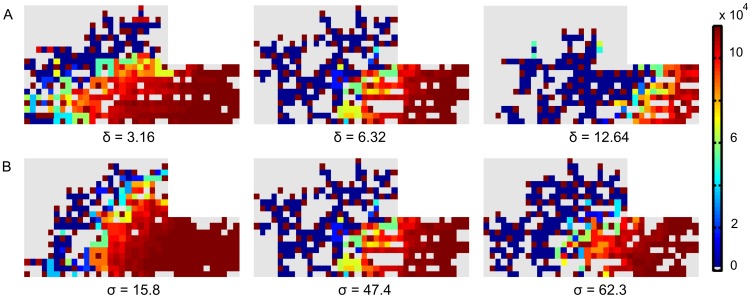
Image of the amount of VEGFR-2 per EC for different Dll4 expression and Notch activity levels at post fracture day 19. The figure is focused on the periosteal callus (area 1 in Figure 3). Remark that the tip cells have a lot of VEGFR-2 (dark brown) whereas the following stalk cells are inhibited, giving rise to a low number of VEGFR-2 (dark blue). (A) Influence of Dll4 levels. (B) Influence of Notch1 activity levels. Left: heterozygous knockout, middle: standard condition, right: overexpression.

Figure 5 shows that the tip cells have high VEGFR-2 levels. The stalk cells are inhibited and have low VEGFR-2 and actin levels. The Dll4-Notch signaling stops when the VEGF-concentration in the callus drops (the VEGFR-2 levels stay constant) (Equations 1–3). The VEGF concentration goes down since the vasculature brings enough oxygen to the fracture site. The endothelial cells far away from the vascular front all have maximal VEGFR-2 levels.

**Figure 6:**
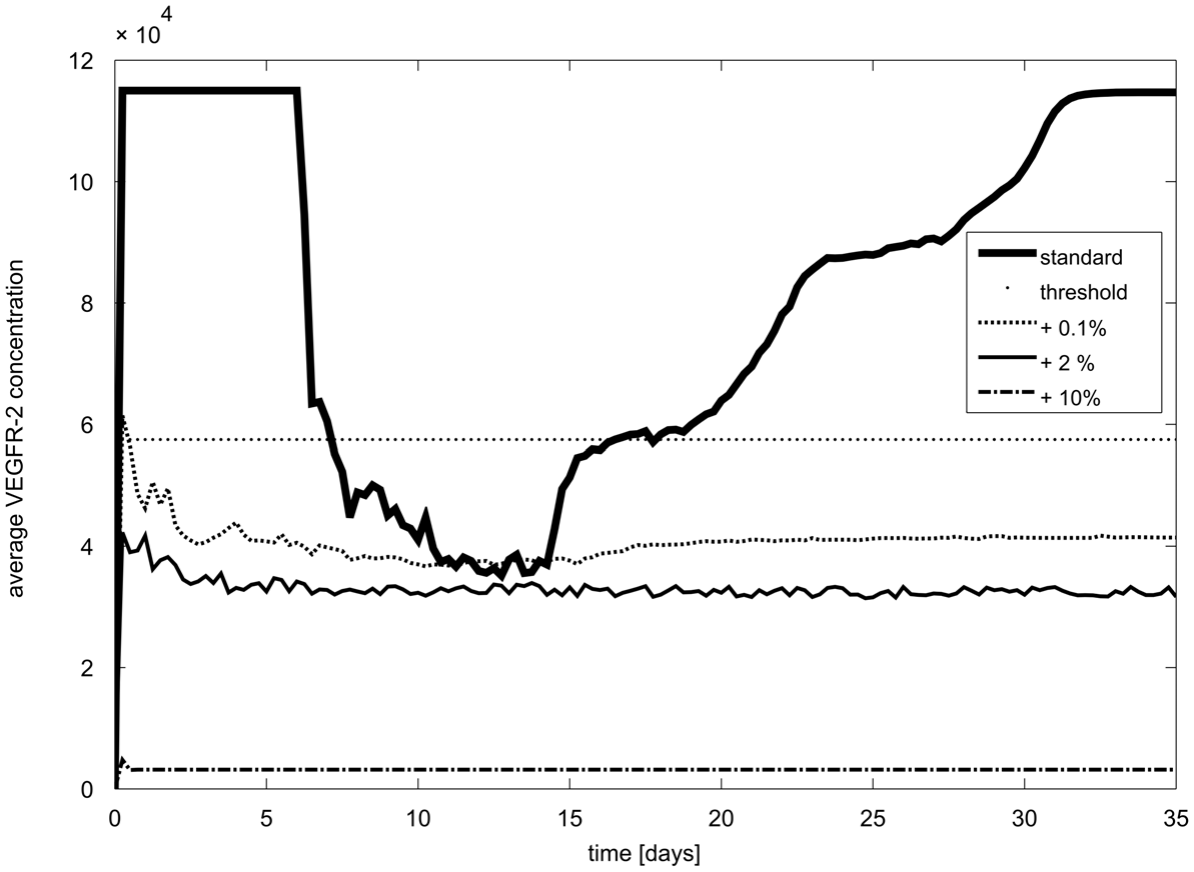
Temporal evolution of the average amount of VEGFR-2 on ECs in the callus for different levels of VEGF addition (corresponding to **Figure 7B**). Evolution of the average VEGFR-2 concentration (day 0 corresponds to the time of fracture). The threshold line represents the first requirement that needs to be fulfilled to obtain the tip cell phenotype, i.e. V>V_max_/2. Remark that in very high VEGF concentrations (+10%) the average receptor concentration is far below the threshold.

The average VEGFR-2 concentration, predicted across all ECs present in the fracture callus, drops at day 7 in the standard condition (Figure 6, standard). Indeed, after 7 days the ECs start to inhibit each other in gaining the tip cell phenotype, resulting in a prediction of enhanced Notch1-signaling and reduction of the average VEGFR-2 levels at the vascular front. At the back, VEGFR-2 levels are predicted to return to their maximal value, which is a direct consequence of Equation 3 (effect of Notch activity on VEFGR-2), and the fact that in the model Notch activity levels of an EC are only governed by VEGF-induced Dll4 expression (in its neighboring cells). As mentioned before, the model only focuses on the lateral inhibition between tip cells and stalk cells through Dll4-Notch. It does not address EC quiescence and the fact that Notch activity in quiescent ECs will be associated with reduced VEGFR-2 receptor levels [25]–[27]. Despite this anomaly in terms of the number of VEGFR-2 receptors, the model correctly predicts highly reduced VEGFR-2 activity levels in quiescent cells (i.e. cells at the back of the vasculature), because of the low VEGF concentrations encountered here. This trend in the average VEGFR-2 concentration (Figure 6, standard) was also measured by Reumann et al. [40]. Reumann et al. characterized the time course of VEGFR-2 mRNA expression during endochondral bone formation in a mouse rib fracture model by quantitative RT-PCR [40]. They observed a small drop (although statistically insignificant) in the median value of VEGFR-2 mRNA expression at three days post fracture [40]. The simulated average VEGFR-2 concentration (Figure 6, standard) follows a similar trend but drops at later time points (day 7 versus day 3). The experimental results [40] were, however, determined in a mouse rib fracture model whereas the parameter values of the model presented in this study were derived from a rat femur fracture model [35]. At the same time, it should be mentioned that Figure 6 represents protein values where Reumann et al. [40] measured mRNA levels. Moreover, Reumann et al. [40] measured the total mRNA content, of all cells present in the callus whereas Figure 6 shows the average VEGFR-2 concentration on the cell membranes of the ECs in the callus. Not only ECs but also osteoblasts and other osteogenic cells express VEGFR-2 [41], which might also explain the temporal difference seen between the experimental and simulated data.

### Impaired angiogenesis

If pharmacological blocking of VEGFR-2 receptors is simulated, the vasculature does not develop since the actin production is inhibited, meaning that the ECs cannot extend filopodia and gain the tip cell phenotype. Due to this impaired vascularization only a small amount of bone is predicted intramembranously, resulting in a non-union between the fractured bone ends.

**Figure 7:**
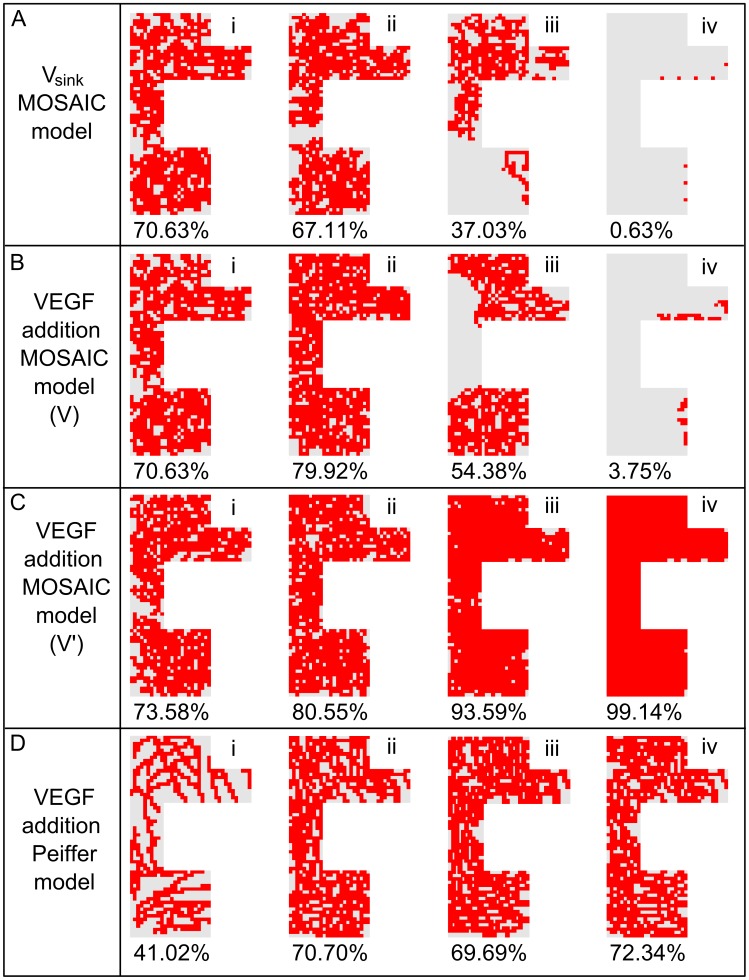
Vasculature at 35 days post fracture for different conditions. (A) Variation in the amount of the decoy receptor VEGFR-1 (i: *V_sink_* = 100% (standard condition), ii: *V_sink_* = 45%, iii: *V_sink_* = 9%, iv: *V_sink_* = 0.9%); (B) Variation in the amount of VEGF addition in the multiscale model with the standard tip cell selection criterion (based on V; Equation 6) (i: 0% (standard condition), ii: 0.1%, iii: 2%, iv: 10%); (C) Variation in the amount of VEGF addition in the multiscale model with an altered tip cell selection criterion (based on V′) (i: 0%, ii: 0.1%, iii: 2%, iv: 10%); (D) Variation in the amount of VEGF addition in the hybrid model (i: 0%, ii: 0.1%, iii: 2%, iv: 10%).

**Figure 8:**
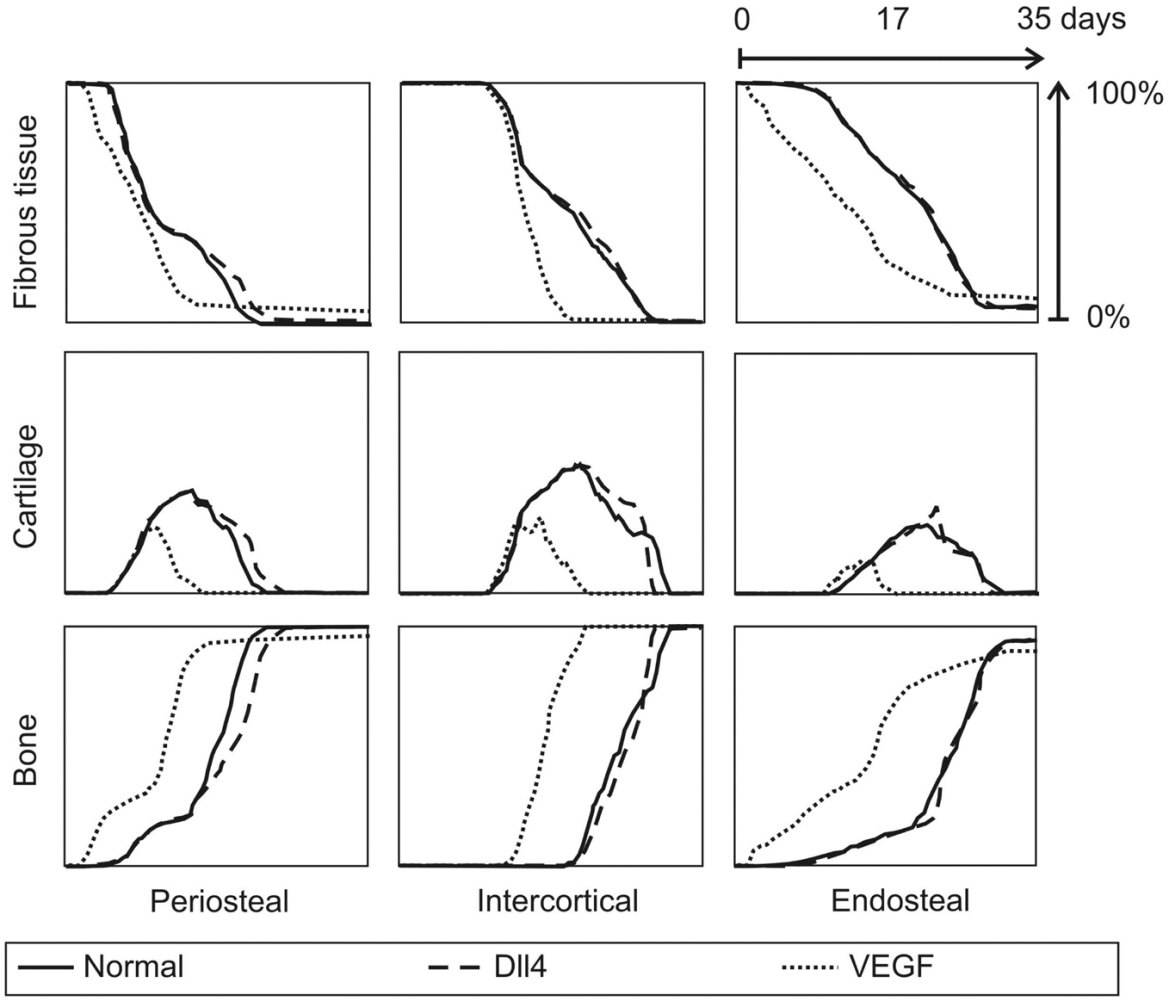
In silico evolution of fracture healing for various conditions. Temporal evolution of the bone, cartilage and fibrous tissue fractions (%) in the periosteal, intercortical and endosteal callus as predicted by the MOSAIC model. (full: normal fracture healing; dashed: Dll4 overexpression (δ = 12.64, Figure 5A); dotted: VEGF-addition (+ 0.1%, Figure 7Bii).

If the VEGF concentration is increased (Figure 7B), the vascular density is initially increased since the VEGFR-2 receptors are being more activated in this stimulating environment (Equation 1). This simulation result is confirmed by many experimental studies that reported an increase in vascularity at the site of VEGF application in a murine femoral fracture healing model [34], a lapine mandibular defect model [42], a murine ectopic model [43] and a rat femoral bone drilling defect model [44]. In the simulations the increase in vascular density leads to faster healing, which is also found experimentally [34], [42] (Figure 8). The MOSAIC model predicts earlier bone formation, less cartilage formation in both the periosteal, intercortical as well as the endosteal callus. Moreover, the cartilage resorption is predicted to be accelerated, another trend which has been reported in experimental studies [43], [44]. It is striking, however, that after 35 days, the MOSAIC model predicts that the VEGF-treated callus contains slightly less bone and more remnants of fibrous tissue than the normal condition (Figure 8).

A further increase of the VEGF concentration (+2%, Figure 7B(iii)) reduces however the vascular density and bone tissue fraction in the MOSAIC model. This is consistent with the trend seen by Street et al., where an optimal dose of VEGF (250 µg) leads to a maximal amount of callus volume (both total and calcified) in a critical rabbit radius segmental gap model [34].

**Figure 9:**
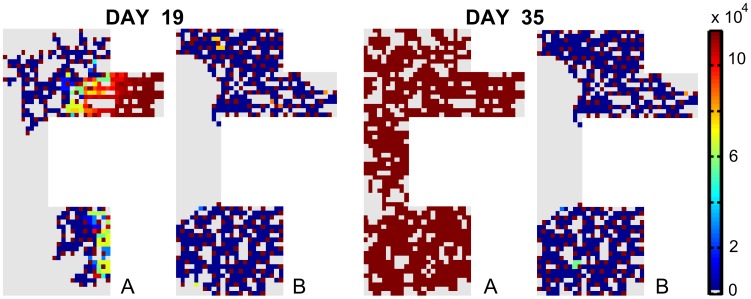
Amount of VEGFR-2 receptors on every endothelial cell. (A) standard condition; (B) addition of 2% VEGF.

In higher VEGF environments (e.g. Figure 7B(iii)) the ECs strongly inhibit each other; creating a salt and pepper pattern of high and low VEGFR-2 levels (Figure 9). In addition, the development of the vasculature ceases after a certain period, as can be seen in Figure 9. The ECs that fulfill the tip cell criteria (Equation 6) will sprout and will initially move perpendicular to the vessel from which it is originating. In case this “mother” vessel is a growing vessel as well that extends towards the source of the chemotactic and haptotactic signals, this implies that the new sprout will initially move perpendicular to the gradients. In this particular configuration (Figure 7B(iii)), the vascular front was progressing in a more “sheet-like” fashion. Consequently, the tip cells persistently want to sprout towards already occupied grid cells, an action that is not allowed in the computational framework. The period after which the vascular development ceases, is shorter in higher VEGF environments. Note that the average VEGFR-2 concentration of all ECs in the callus reaches a constant level which is reduced in high VEGF environments (Figure 6).

When a reduction of VEGF concentration by means of the addition of VEGF-antibodies is simulated, results demonstrate that the VEGFR-2 receptor is not sufficiently stimulated. This leads to an impaired vasculature and a non-union between the fractured bone ends (see Table 3, Equation 1). The simulated reduction in vascular density is consistent with the experimental findings of Street et al. who incorporated a fracture hematoma supernatant with neutralizing monoclonal antibody to human VEGF in a Matrigel vehicle, which was then implanted in a murine dorsal wound model [45]. There was a significant decrease in the number of blood vessels formed in the Matrigel vehicle with neutralizing monoclonal antibody when compared to the fracture hematoma supernatant alone [45].

**Table 3:** Overview of the results of the sensitivity analysis.

Condition		Week 1	Week 3	Week 5
**standard**		**2.34%**	**46.25%**	**70.63%**
***σ***	33%	3.05%	56.25%	78.20%
	**100%**	**2.34%**	**46.25%**	**70.63%**
	133%	2.19%	45.00%	68.91%
***δ***	50%	2.73%	54.92%	75.23%
	**100%**	**2.34%**	**46.25%**	**70.63%**
	200%	2.57%	37.66%	64.61%
***V_sink_***	364%	5.00%	48.75%	70.08%
	**100%**	**2.34%**	**46.25%**	**70.63%**
	45%	0.63%	39.92%	67.11%
	9%	0.63%	15.86%	37.03%
	0.9%	0.63%	0.63%	0.63%
V′*	25%	3.20%	67.34%	76.64%
	**100%**	**2.34%**	**46.25%**	**70.63%**
	400%	0.63%	38.13%	56.56%
	750%	0.63%	20.00%	51.25%
	1000%	0.63%	15.16%	35.31%
***A_0_***	0%	2.42%	46.95%	72.73%
	50%	2.42%	46.95%	72.73%
	**100%**	**2.34%**	**46.25%**	**70.63%**
***D_0_***	**0%**	**2.34%**	**46.25%**	**70.63%**
	28%	2.34%	46.25%	70.63%
	64%	2.34%	46.25%	70.63%
	100%	2.34%	46.25%	70.63%
**VEGF addition**	0.1%	36.02%	76.09%	79.92%
	2%	28.98%	54.22%	54.38%
	2.4%	16.64%	16.64%	16.64%
	10%	3.75%	3.75%	3.75%
**VEGF removal**	−1%	0.63%	10.70%	36.56%
	−1.5%	0.63%	4.84%	36.64%
	−3%	0.63%	0.78%	0.78%
	−5%	0.63%	0.63%	0.78%

The vascular density is measured at three time points post fracture (7, 21 and 35 days). In the standard condition the parameter values are *σ* = 47.4, *δ* = 6.32, *V_sink_* = 0.275, V′* = 200, *A_0_* = 5000, *D_0_* = 0, which can also be found in Tables 1 and 2.

### Sensitivity analysis

The sensitivity analysis indicates that the model results are greatly influenced by the parameters *σ*, *δ* and *V_sink_* and that the MOSAIC model is insensitive to the initial conditions of Dll4 (*D_0_*) and actin (*A_0_*). An overview of the results of the sensitivity analysis can be found in Table 3.

Simulations of the heterozygous knockout genotypes (*δ* = 50%, *σ* = 33%) show an increased sprouting due to a clearly reduced inhibition of the tip cell phenotype (Equations 2–3). Figure 5 demonstrates that the endothelial cells behind the brown (tip) cells with high VEGFR-2 levels are strongly inhibited in the normal case but are weakly inhibited in the simulated knockout. The overexpression of Dll4 (*δ* = 200%) increases the inhibition of the tip cell phenotype resulting in a decrease of the vascular density (Table 3) and a delay in the endochondral bone formation process, particularly in the periosteal callus (Figure 8). The increase in *σ* also causes a more potent suppression of the tip cell phenotype in the stalk cells, due to an increased down-regulation of VEGFR-2 by Notch1 (Equation 3). In turn, this leads to a decrease of the vascular density (see Figure 5B, Table 3). Thus, if the tip cell phenotype is more inhibited (by increasing *δ*, which defines the enhancement of Dll4 expression due to VEGFR-2 activation (Equation 2) or increasing *σ*, which represents the inhibition of the VEGFR-2 expression due to Notch1 activation (Equation 3)), the vascular density is reduced. This simulation result corresponds to experimental observations [5], [46], [47]. Hellström et al. [5] showed that the inhibition of Notch signaling (by inhibiting Notch receptor cleavage and signaling with γ-secretase inhibitors, by heterozygous inactivation of the Notch ligand Dll4 or by endothelial cell specific deletion of Notch1) promotes an increase in the number of tip cells in the retina of newborn mice. Conversely, a 35% decrease in filopodia density and a 45% decrease in vessel density were found in a direct gain of function experiment of the Notch1 receptor.

Simulating an increase of the decoy receptor VEGFR-1 (by decreasing *V_sink_*) results in a reduction of the vascular density since less VEGF remains available for VEGFR-2 activation (see Figure 7A, Equation 1). This is consistent with Flt-1 (VEGFR-1) loss- and gain-of-function data in zebrafish embryos [34], [48]. Moreover, Street et al. showed that Flt-IgG treatment decreased the vascularity by 18% and impaired cortical bone defect repair in a murine femoral fracture healing model [34]. Similarly, the *in silico* results show a delayed or even impaired healing of the fracture due to the reduced vascular density. In Figure 7A(iv) the VEGF concentration is too low to activate the VEGFR-2 receptors which stops the angiogenic process. Since only a small amount of bone will be formed intramembranously in the fracture callus of Figure 7A(iv), the impaired vascularization will result in a non-union.

If the threshold of VEGFR-2 activation V′*, below which the actin production and tip cell movement is inhibited, is increased, the vascular density decreases (see Table 3). The initial amount of actin (*A_0_*) only slightly influences the final vascular density (see Table 3). The other variables related to the fracture healing, were not influenced. Similarly, the final vascular density is insensitive to the initial intracellular amount of Dll4 (*D_0_*) (see Table 3).

## Discussion

This study established a novel multiscale model of angiogenesis in the context of fracture healing, by integrating an agent-based model of tip cell selection [3] into a previously developed hybrid model of fracture healing [18]. The bone regeneration process was predicted by the MOSAIC model in accordance with experimental reports and previously validated *in silico* results [18]. The MOSAIC model was also able to capture many experimentally observed aspects of tip cell selection: the salt and pepper pattern seen in developing vascular structures under normal angiogenic conditions, i.e. a tip cell with high VEGFR-2 and actin levels followed by a stalk cell characterized by strong Notch1 signaling and therefore reduced VEGFR-2 and actin levels [30], an increased tip cell density and a higher vascular density in case of Dll4 heterozygous knockouts [5] and an excessive number of tip cells (leading to a very high vascular density) in high VEGF concentrations [3], [34]. The sensitivity analysis also indicated the most influential parameters of the MOSAIC model (*δ*, *σ* and *V_sink_*).

This study has addressed some, but not all of the limitations of the Peiffer-model [18]. In the MOSAIC model the tip cell selection is based on Dll4/Notch1 signaling whereas the Peiffer-model [18] implemented sprouting with phenomenological rules such that
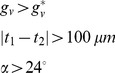
(9)i.e. in the Peiffer-model the VEGF concentration needs to be high enough (10 ng/ml), there needs to be a minimal separation of 100 µm between two tip cells and the movement direction of the new tip cell should make an angle of >24° with the orientation of its mother vessel. Moreover, the Peiffer-model foresees three healing days between subsequent sprouting events, which has no experimental foundation and has been removed in the MOSAIC model. Consequently, the MOSAIC model is more mechanistic, allowing investigation of different mutant and druggable cases in the signaling pathways, leading to real predictions for experimentation, which was not possible in the Peiffer-model. Since the incorporation of the lateral inhibition mechanism leads to a denser plexus in the MOSAIC model than in the Peiffer-model, we have reduced the oxygen production rate by a factor of two so that the final oxygen concentrations are the same in both models. Experimental results show that the vascular plexus is indeed very dense in the fracture callus [36], [37].

In the MOSAIC model the tip cell velocity increases with the active VEGFR-2 levels, indicating that both the level of VEGFR-2 and the external VEGF-concentration influence the tip cell speed. This is consistent with the experimental data of Arima et al. [49]. They used time-lapse imaging in a murine aortic ring assay (with and without VEGF) to quantify the behavior of the endothelial cells during angiogenic morphogenesis [49]. Arima et al. reported that VEGF-induced vessel elongation was only due to greater displacement per tip cell [49]. Moreover, treatment with Dll4-antibodies also resulted in a greater displacement per tip cell [49]. This is due to the reduced inhibitory actions of Dll4, causing a greater number of cells to have high VEGFR-2 levels. These results are however contested by Jakobsson et al. who quantified the average migration speed of wild-type (DsRed and YFP) and heterozygous Vegfr2^+/egfp^ endothelial cells in different chimaeric embryoid bodies [50]. They observed no difference in migration speed, indicating that VEGFR-2 levels do not determine EC migration velocity [50]. Clearly, more research is necessary to elucidate the above observations and improve the current implementation of tip cell migration in future versions of the model.

The MOSAIC model indicates a key role of the decoy receptor VEGFR-1 (modeled via *V_sink_*) (Equation 1). Increasing the amount of VEGFR-1, results in a decrease of the vascular density (Figure 7A) which is also seen in loss- and gain-of-function data [34], [48]. Both the MOSAIC model and the model of Bentley et al. [3], use a constant value to represent the decoy-effect of the VEGFR-1 receptor. There is however experimental evidence that both VEGFR-1 and its soluble form are up-regulated in Notch-activated stalk cells [4], [51]. Hence, the stalk cells phenotype is not only consolidated by a decrease in VEGFR-2 but also by an increase in the competing VEGFR-1 receptor. The results of Krueger et al. also suggest that VEGFR-1 regulates tip cell formation in a Notch-dependent manner [48].

**Figure 10:**
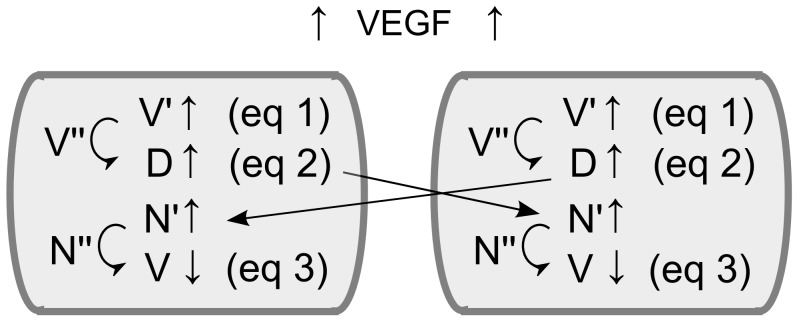
Schematic representation of the Dll4-Notch pathway in high VEGF environments for two neighboring ECs. Remark that although the VEGFR-2 receptor is down-regulated, the VEGF concentration is high enough to compensate for this effect resulting in high *V′* levels (Equation 1).

The MOSAIC model displays interesting behavior in high VEGF environments (see Figure 7). Initially, the increase in VEGF has a positive effect, resulting in a very dense vasculature since the VEGFR-2 receptors are being more activated in this stimulating environment (+0.1%; see Equation 1). This leads to a faster healing, which is also found experimentally [34], [42]. A further increase (+2%), however, reduces the vascular density in the MOSAIC model. This is consistent with the trend seen by Street et al. [34]. Note that a salt and pepper pattern of high and low VEGFR-2 levels is created and maintained in high VEGF environments (+2%; Figure 7B(iii) and Figure 9). To explain this observation, one needs to look at the beginning of the angiogenic process in the fracture callus. Initially, some endothelial cells gain the tip cell phenotype and start to migrate. Gradually sprouts arise in the developing vasculature which increases the network size and alters the local VEGF-levels due to the influence of the oxygen tension on VEGF-production. The original tip cells maintain their advantage (e.g. located in a higher VEGF environment) by strongly inhibiting their neighboring ECs, creating a salt and pepper pattern of VEGFR-2 levels. In standard conditions the vasculature would start to mature, leading to quiescent ECs. In high VEGF environments (+2%), however, this salt and pepper pattern of VEGFR-2 is maintained (Figure 9), illustrating that some ECs have (very) high VEGFR-2 levels leading to a persistent inhibition of their neighboring ECs (characterized by low VEGFR-2 levels). Figure 6 shows that these high VEGFR-2 levels are cancelled out by the low VEGFR-2 levels resulting in a “steady state” level of the average VEGFR-2 concentration. In high VEGF environments (+2%, +10%) this “steady state” level is gradually reduced (Figure 6), implying the dominance of the lower VEGFR-2 levels. Mathematically, this result follows from Equations (1) and (3), indicating that in high VEGF concentrations both the active VEGFR-2 (*V′*) and Notch (*N′*) (and with a delay the effective active VEGFR-2 (*V″*) and Notch (*N″*)) are high, resulting in a reduction of the VEGFR-2 receptor (Figure 10). Consequently, the average VEGFR-2 concentration is reduced below the threshold for tip cell formation (Figure 6). In other words, the majority of the ECs have too little VEGFR-2 receptors to assume the tip cell phenotype. In the extreme case, this finally results in the inhibition of the development of the vasculature since there are no tip cells to lead the sprouts towards the VEGF source (Figure 7B(iv)).

Interestingly, Figure 7D shows that similar results cannot be obtained with the Peiffer-model [18], i.e. the vascular density is not reduced in high VEGF environments (+2%, +10%). This is due to the phenomenological rules that determine the tip cell selection in the Peiffer-model (Equation 9). A similar “non-linear” EC response to VEGF concentrations would only be possible with the Peiffer-model if another phenomenological rule would be implemented that e.g. down-regulates tip cell selection at high VEGF responses. In contrast, the “non-linear” response follows naturally from the mechanistic rules of tip cell selection that were implemented in the MOSAIC model. That is, the down-regulation of the tip cell selection in high VEGF environments (+2%, +10%) arises from the negative feedback loop in the Notch-Dll4 signaling pathway. Moreover, in high VEGF-environments (+10%) and at the back of the developing vasculature, we see an indication that patches of endothelial cells oscillate between cell fates (switching between high and low VEGFR-2 levels). These patches are also predicted by the model of Bentley et al. and are observed during pathological angiogenesis [3]. In the future, we will further investigate the conditions that give rise to these oscillations and their implications on the development of the vasculature.

The results of the MOSAIC model are based on the assumption that a tip cell phenotype can only be acquired if the levels of VEGFR-2 and actin are sufficiently high (Equation 6). If this criterion was changed by replacing the requirement on VEGFR-2 by a similar requirement for the level of active VEGFR-2, some ECs could become tip cells, since *V′* is high in high VEGF environments (+2%, +10%), and the vasculature would fully develop (Figure 7C). These results show the added value of the MOSAIC model: the intracellular module and its related state variables and rules decide on the EC response to the extracellular VEGF environment, in turn determining the healing response at the tissue level (Figure 8). In case the criterion for tip cell selection is specified in terms of VEGFR-2 (and actin) the absence of blood vessel formation will result in a non-union or a delayed union of the fracture. However, when this criterion is replaced by one that relies on the levels of active VEGFR-2 (and actin), a vascular and healing response is retrieved, similar to the Peiffer-model.

Clearly, these findings give rise to some interesting biological questions on a proper criterion for the tip cell phenotype. Since the VEGFR-2 levels are strongly reduced in high VEGF environments (+10%), the tip cells lose their tip cell phenotype and stop migrating although there is a strong angiogenic signal present. Can tip cells move in high VEGF environments although they do not have enough VEGFR-2 receptors? If so, should the tip cell criterion (Equation 6) be based on the active VEGFR-2 levels (*V′*), since these remain high in high VEGF concentrations? Or is the down-regulation of VEGFR-2 receptors in high VEGF environments compensated by other signaling cascades that have VEGFR-2 as one of their downstream targets (leading to an increase of VEGFR-2)?

In this study, the model of Peiffer et al. [18] was combined with a detailed model of Dll4-Notch1 signaling [3]. Some simplifications were however made to the model of Bentley et al. due to computational reasons, i.e. the size and shape of the ECs are fixed in the PDE framework of the MOSAIC model. Consequently, every EC is represented by one agent whereas Bentley et al. use a varying amount of membrane agents for every EC [3]. This does not only allow Bentley et al. to model the change in membrane and cell shape in great detail, but also to include cellular polarity (non-uniform distribution of receptors and ligands across the cell membrane and cell-cell junctions). In the MOSAIC model, filopodia extension is modeled implicitly by an increase in the level of the “actin” variable upon VEGFR-2 activation. This is consistent with current knowledge that activation of Cdc42 by VEGF triggers filopodia formation [4]. Bentley et al. modelled filopodia extension in more detail by adding membrane agents to the cellular membrane. As a result, the number of VEGFR-2 receptors will alter due to filopodia extension, which is proposed to be a mechanism to consolidate the tip cell fate [3]. In the MOSAIC model the accumulation of actin does not lead to an increase in the amount of VEGFR-2 levels or to a change in the microenvironmental range that can be probed by the tip cell. However, if the molecular mechanisms of filopodia extension and its implications on probing the environment and the directionality of tip cell movement are clearer, these can be readily incorporated in the multiscale framework.

The mechanism of lateral inhibition is based on Dll4/Notch1 signaling between the endothelial cells of the developing sprout. Delta-Notch signaling is however an evolutionary conserved pathway that is also involved in cell fate specification, tissue patterning and morphogenesis [2], . In angiogenesis specifically, Notch signaling influences endothelial cell specification [4], [5], [30], [54], [55], endothelial proliferation [29], [30], cell migration [2], [30], filopodia formation [30], cell adhesion [30], and post-angiogenic vessel remodeling and endothelial cell quiescence [56]. These effects are not only dependent on Dll4 and Notch1 but also on the other ligands (Delta-like 1, Delta-like 3, Jagged-1 and Jagged-2) and receptors (Notch2, Notch3 and Notch4) [57]. Due to the complexity and interdependency of these pathways, only the influence of Dll4-Notch1 signaling on tip cell selection was modeled. Consequently, in the model once the VEGF levels are reduced due to the restoration of the blood flow and tissue oxygenation, the Dll4-Notch1 signaling pathway is not active anymore. This is predicted to occur in the ECs that are located at the back of the vasculature, returning their VEGFR-2 levels to the maximal value. As mentioned before this contradicts the fact that in quiescent cells VEGFR-2 levels will be minimal and smaller than those of migrating cells, which is consistent with high Notch activity in quiescence [25]–[27]. Since the MOSAIC model does not include the role of Notch in quiescence, the simulation results are only accurate for the initial formation of the vasculature and not for the maturation and stabilization of the vascular plexus. This does not, however, alter the main findings of this work concerning sprouting angiogenesis.

Besides VEGFR-2, also other VEGF receptors, such as VEGFR-1 and VEGFR-3 play a role in angiogenesis. Although the VEGFR-3 receptor is mainly active in lymphangiogenesis, recent experimental evidence indicates that VEGFR-3 is up-regulated in tip cells during pathological angiogenesis [4], [58]. Blocking this receptor reduces the amount of sprouting and EC proliferation. It appears that VEGFR-2 induces VEGFR-3 expression in tip cells, whereas it is down-regulated in stalk cells by Notch [4], [59]. However, when more quantitative experimental data become available on the role of VEGFR-1 and VEGFR-3 in sprouting angiogenesis, this can be incorporated in the MOSAIC model.

The MOSAIC model only focuses on soluble VEGF, whereas VEGF-isoforms that bind to the extracellular matrix are essential to establish the VEGF gradients required for guided tip cell migration [60]. Some modeling work has already been done in this area [61], [62], e.g. Vempati et al. used a detailed molecular model of VEGF ligand-receptor kinetics and transport to investigate the VEGF-isoform specific spatial distributions observed experimentally [61]. Many other factors, such as neuropilin 1 (NRP-1), fibroblast growth factor (FGF), and platelet-derived growth factor (PDGF) regulate the angiogenic response as well [2]. Nevertheless, it has been stated repeatedly that VEGF is “the principal dancer” during angiogenesis [29],[30].

The proposed MOSAIC model incorporates biological processes at various temporal and spatial scales: an intracellular module that includes Dll4/Notch1 signaling to determine tip cell selection, a discrete representation of the ECs allowing an accurate representation of the developing vascular network and a continuum description of oxygen, growth factors and tissues that finally result in the healing of the fracture by the formation of bone. Our simulation results demonstrate the advantages of such a multiscale approach. Firstly, the interplay between molecular signals, in particular VEGF, Dll4 and Notch1, endothelial cell phenotypic behavior and bone formation was explored. In this way, the MOSAIC model could be used to verify to what extent gene knockouts, injection of VEGF-antibodies or blockage of VEGF-receptors leads to a “bone phenotype” in terms of rate and amount of bone formation (see e.g. Figure 8). While some of these simulation results could be (qualitatively) compared to experimental data, it is clear that future research efforts must be focused on a more comprehensive quantitative validation. Again, the multiscale nature of the simulation results presents an advantage here, as it allows for a validation at different scales (molecular, cellular and tissue scale). Secondly, the proposed multiscale model is more mechanistic since tip cell selection is based on intracellular dynamics (Dll4-Notch1 signaling), rather than the phenomenological rules that were used in Peiffer et al. [18]. As such, the MOSAIC model enabled to extend the model of Bentley et al. [3] to the context of fracture healing, leading to interesting emergent behavior at the macro-scale. More specifically, whereas the Peiffer-model predicts the presence of a vascular network in high VEGF environments (+10%) the MOSAIC model (depending on the tip cell criterion) predicts the absence of a vascular network (see Figure 7), which was a direct consequence of the Dll4-Notch feedback mechanism (see explanation related to Figure 10). In conclusion, the proposed multiscale method was found to be a useful tool to investigate possible biological mechanisms across different time and spatial scales, thereby contributing to the fundamental knowledge of sprouting angiogenesis and its relation to fracture healing.

## Supporting Information

Text S1 This file contains a detailed description of the mathematical model used in this study including the full set of equations, parameter values, boundary and initial conditions and implementation details.
(DOC)
