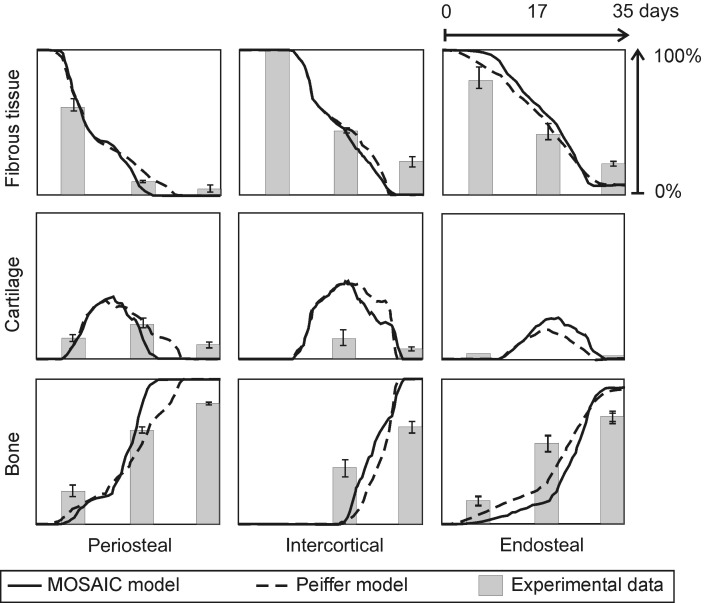# Correction: MOSAIC: A Multiscale Model of Osteogenesis and Sprouting Angiogenesis with Lateral Inhibition of Endothelial Cells

**DOI:** 10.1371/annotation/38264a13-d4b5-49cd-b54e-47330bb19fe9

**Published:** 2013-03-28

**Authors:** Aurélie Carlier, Liesbet Geris, Katie Bentley, Geert Carmeliet, Peter Carmeliet, Hans Van Oosterwyck

The published version of Figure 4 was the original, rather than the revised version. Please see the correct Figure 4 here: 

**Figure:**