# Supplementary material for: MOSAIC: A Multiscale Model of Osteogenesis and Sprouting Angiogenesis with Lateral Inhibition of Endothelial Cells
Source: PLoS Comput Biol. 2012 Oct 11;8(10):e1002724. doi: 10.1371/journal.pcbi.1002724 (PMC3469420; doi:10.1371/journal.pcbi.1002724)
Supplement: Text S1 — This file contains a detailed description of the mathematical model used in this study including the full set of equations, parameter values, boundary and initial conditions and implementation details. (DOC) [file pcbi.1002724.s001.doc]

**Supporting information**

***Continuum model***

The following **equations** describe the spatiotemporal evolution of the densities of mesenchymal stem cells (*c_m_*), fibroblasts (*c_f_*), chondrocytes (*c_c_*), osteoblasts (*c_b_*), endothelial cells (*c_v_*), fibrous tissue (*m_f_*), cartilage (*m_c_*), bone (*m_b_*) and vascular matrix (*m_v_*) and the concentrations of chondrogenic (*g_c_*), osteogenic (*g_b_*) and vascular growth factors (*g_v_*) and concentration of oxygen, constituting the tissue-level model applied in this study:

$\frac{\partial c_{m}}{\partial t}=\nabla\left[D_{m}\nabla c_{m}-C_{m_{CT}}c_{m}\nabla(g_{b}+g_{v})-C_{m_{HT}}c_{m}\nabla m\right]+A_{m}c_{m}\left[1-\alpha_{m}c_{m}\right]-F_{1}c_{m}-F_{2}c_{m}-F_{4}c_{m}$ (1)

$\frac{\partial c_{f}}{\partial t}=\nabla\left[D_{f}\nabla c_{f}-C_{f}c_{f}\nabla g_{b}\right]+A_{f}c_{f}\left[1-\alpha_{f}c_{f}\right]+F_{4}c_{m}-F_{3}d_{f}c_{f}$ (2)

$\frac{\partial c_{c}}{\partial t}=A_{c}c_{c}\left[1-\alpha_{c}c_{c}\right]+F_{2}c_{m}-F_{3}c_{c}$ (3)

$\frac{\partial c_{b}}{\partial t}=A_{b}c_{b}\left[1-\alpha_{b}c_{b}\right]+F_{1}c_{m}+F_{3}c_{c}-d_{b}c_{b}$ (4)

$\frac{\partial m_{f}}{\partial t}=P_{fs}\left(1-\kappa_{f}m_{f}\right)c_{f}-Q_{f}m_{f}m_{c}c_{b}$ (5)

$\frac{\partial m_{c}}{\partial t}=P_{cs}\left(1-\kappa_{c}m_{c}\right)c_{c}-Q_{c}m_{c}c_{b}$ (6)

$\frac{\partial m_{b}}{\partial t}=P_{bs}\left(1-\kappa_{b}m_{b}\right)c_{b}$ (7)

$\frac{\partial g_{c}}{\partial t}=\nabla\left[D_{gc}\nabla g_{c}\right]+E_{gc}c_{c}-d_{gc}g_{c}$ (8)

$\frac{\partial g_{b}}{\partial t}=\nabla\left[D_{gb}\nabla g_{b}\right]+E_{gb}c_{b}-d_{gb}g_{b}$ (9)

$\frac{\partial g_{v}}{\partial t}=\nabla\left[D_{gv}\nabla g_{v}\right]+E_{gvb}c_{b}+E_{gvc}c_{c}-g_{v}\left(d_{gv}+d_{gvc}c_{v}\right)$ (10)

$\frac{\partial n}{\partial t}=\nabla\left[D_{n}\nabla n\right]+E_{n}c_{v}-d_{n}n$ (11)

The migration of mesenchymal stem cells is a combination of random and directed motion [1]. The random motion was modelled as a haptokinetic process. Random motion is influenced by the total matrix density, defined as $m=m_{f}+m_{c}+m_{b}+m_{v}$, such that in the absence or abundance of extracellular matrix cells cease to move.

$D_{m}=\frac{D_{hm}m}{K_{hm}^{2}+m^{2}}$ (12)

Chemotaxis was modelled using a receptor-kinetic form, giving a maximum chemotactic response at a particular growth factor concentration [2]. The chemotactic response of mesenchymal stem cells depends both on osteogenic and angiogenic growth factors.

$\begin{matrix} C_{m_{CT}}=\frac{C_{kCTm}(g_{b}+g_{v})}{K_{kCTm}^{2}+(g_{b}+g_{v})^{2}} \\ C_{f}=\frac{C_{kf}g_{b}}{K_{kf}^{2}+g_{b}^{2}} \end{matrix}$ (13)

The haptotactic coefficient was taken from [3], based on a kinetic analysis of a model mechanism for the cell-surface-receptor-extracellular-ligand binding dynamics [4].

$C_{m_{HT}}=\frac{C_{kHTm}}{\left(K_{kHTm}+m\right)^{2}}$ (14)

The proliferation of stem cells, as well as the other four cell types, is modelled by a logistic growth function, whereby the proliferation rate depends on the surrounding matrix density [3,5].

$A_{i}=\frac{A_{i0}m}{K_{i}^{2}+m^{2}} \text{for} i=m,f,c,b$ (15)

The differentiation of mesenchymal stem cells towards osteoblasts is mediated by the presence of osteogenic and angiogenic growth factors [6]. For high chemical concentrations, a saturation effect was modelled to take place [7].

$F_{1}=\left(\frac{Y_{11}g_{b}}{\left(H_{11}+g_{b}\right)}+\frac{Y_{11}g_{v}}{\left(H_{12}+g_{v}\right)}\right).\frac{Y_{12}n^{6}}{\left(I_{v}^{6}+n^{6}\right)}$ (16)

A similar function, depending on the concentration of the chondrogenic growth factor, was used to model the differentiation towards chondrocytes.

$F_{2}=\frac{Y_{2}g_{c}}{H_{2}+g_{c}}$ (17)

The following function describes the endochondral replacement of chondrocytes [8,9]:

$F_{3}=\frac{m_{c}^{6}}{\left(B_{ec}^{6}+m_{c}^{6}\right)}\cdot\frac{Y_{3}g_{b}}{\left(H_{3}+g_{b}\right)}\cdot\frac{n^{6}}{\left(B_{v}^{6}+n^{6}\right)}$ (18)

The production of chondrogenic growth factors occurs up to a certain saturation concentration, after which the production rate levels off. The production is also dependent on the matrix density:

$E_{gc}=\frac{G_{gc}g_{c}}{\left(H_{gc}+g_{c}\right)}\cdot\frac{m}{\left(K_{gc}^{3}+m^{3}\right)}$ (19)

The production and decay of osteogenic growth factor were modelled in a similar way, except that the osteogenic growth factor production rate is not limited by matrix density.

$E_{gb}=\frac{G_{gb}g_{b}}{H_{gb}+g_{b}}$ (20)

Angiogenic growth factors are produced both by osteoblasts and hypertrophic chondrocytes. The production rate is proportional to the present growth factor concentration (up to a saturation level) and also saturates for high angiogenic growth factor concentrations, leading to the following function for the production rates by osteoblasts $E_{gvb}$ and hypertrophic chondrocytes $E_{gvc}$:

$E_{gvb}=\frac{G_{gvb}H_{gv}^{6}}{\left(H_{gv}^{6}+g_{v}^{6}\right)}\cdot\frac{K_{n}^{6}}{\left(K_{n}^{6}+n^{6}\right)}$ (21)

$E_{gvc}=\frac{G_{gvc}H_{gv}^{6}}{\left(H_{gv}^{6}+g_{v}^{6}\right)}\cdot\frac{K_{n}^{6}}{\left(K_{n}^{6}+n^{6}\right)}.\frac{m_{c}^{6}}{\left(B_{ec}^{6}+m_{c}^{6}\right)}$ (22)

The release of oxygen by the blood vessels is modeled according to:

$E_{n}=\frac{G_{n}H_{n}^{6}}{H_{n}^{6}+n^{6}}$ (2”)

The following **scalings** were chosen for the non-dimensionalisation of the variables:

$$\tilde{m}_{b}=\frac{m_{b}}{m_{0}}, \tilde{g}_{c}=\frac{g_{c}}{g_{0}}, \tilde{g}_{b}=\frac{g_{b}}{g_{0}}, \tilde{g}_{v}=\frac{g_{v}}{g_{0}}, \tilde{n}=\frac{n}{n_{0}}$$

Typical time and length scales for fracture healing in rodent studies are $T$ = 1 day and $L$ = 3.5 mm [10]. A representative concentration of the collagen content in the tissues under investigation is $m_{0}$ = 0.1 g/ml. Typical growth factor concentrations are in the order of magnitude of $10^{-9}$ M (mol/l) [11,12]. Taking into account the order of magnitude of the molecular weight of the growth factors (100 kDa = 100 kg/mol), this results in a non-dimensionalisation value of $g_{0}$ = 100 ng/ml. Based on geometrical constraints, a typical value for cell density at the beginning of the healing process was taken to be: $c_{0}$ = $10^{6}$ cells/ml [7]. A typical value for n_0_ was chosen to be 39 mol/m^3^.

The parameter values were derived from literature where possible and estimated when no relevant data was available. We refer to Geris et al. [13] for a detailed description of the parameter derivation and estimation. The parameters were **non-dimensionalised** as follows (tildes referring to non-dimensionalised values):

$$\tilde{D}_{hm}=\frac{D_{hm}T}{L^{2}m_{0}}, \tilde{K}_{hm}=\frac{K_{hm}}{m_{0}}, \tilde{C}_{kCTm}=\frac{C_{kCTm}T}{L^{2}}, \tilde{K}_{kCTm}=\frac{K_{kCTm}}{g_{0}}, \tilde{C}_{kHTm}=\frac{C_{kHTm}T}{L^{2}m_{0}}, \tilde{K}_{kHTm}=\frac{K_{kHTm}}{m_{0}}$$

$$\tilde{A}_{m0}=\frac{A_{m0}T}{m_{0}}, \tilde{K}_{m}=\frac{K_{m}}{m_{0}}, \tilde{Y}_{11}=Y_{11}T, \tilde{H}_{11}=\frac{H_{11}}{g_{0}}, \tilde{Y}_{12}=Y_{12}T, \tilde{H}_{12}=\frac{H_{12}}{g_{0}}, \tilde{Y}_{2}=Y_{2}T, \tilde{H}_{2}=\frac{H_{2}}{g_{0}}, \tilde{F}_{4}=F_{4}T$$

$$\tilde{D}_{f}=\frac{D_{f}T}{L^{2}}, \tilde{C}_{kf}=\frac{C_{kf}T}{L^{2}}, \tilde{K}_{kf}=\frac{K_{kf}}{g_{0}}, \tilde{A}_{f0}=\frac{A_{f0}T}{m_{0}}, \tilde{K}_{f}=\frac{K_{f}}{m_{0}}, \tilde{Y}_{5}=Y_{5}T, \tilde{H}_{5}=\frac{H_{5}}{m_{0}}, \tilde{B}_{v}=\frac{B_{v}}{m_{0}}, \tilde{B}_{ec}=\frac{B_{ec}}{m_{0}}$$

$$\tilde{Y}_{3}=Y_{3}T, \tilde{H}_{3}=\frac{H_{3}}{g_{0}}, \tilde{d}_{f}=d_{f}, \tilde{A}_{c0}=\frac{A_{c0}T}{m_{0}}, \tilde{K}_{c}=\frac{K_{c}}{m_{0}}, \tilde{C}_{kb}=\frac{C_{kb}T}{L^{2}}, \tilde{K}_{kb}=\frac{K_{kb}}{g_{0}}, \tilde{A}_{b0}=\frac{A_{b0}T}{m_{0}}, \tilde{K}_{b}=\frac{K_{b}}{m_{0}}$$

$$\tilde{d}_{b}=d_{b}T, \tilde{P}_{fs}=\frac{P_{fs}Tc_{0}}{m_{0}}, \tilde{Q}_{f}=Q_{f}m_{0}c_{0}T, \tilde{P}_{cs}=\frac{P_{cs}Tc_{0}}{m_{0}}, \tilde{Q}_{c}=Q_{c}c_{0}T, \tilde{P}_{bs}=\frac{P_{bs}Tc_{0}}{m_{0}}, \tilde{D}_{gc}=\frac{D_{gc}T}{L^{2}},$$

$$\tilde{G}_{gc}=\frac{G_{gc}Tc_{0}}{g_{0}m_{0}^{2}}, \tilde{H}_{gc}=\frac{H_{gc}}{g_{0}}, \tilde{K}_{gc}=\frac{K_{gc}}{m_{0}}, \tilde{d}_{gc}=d_{gc}T, \tilde{D}_{gb}=\frac{D_{gb}T}{L^{2}},\tilde{I}_{v}=\frac{I_{v}}{m_{0}}, \tilde{G}_{gb}=\frac{G_{gb}Tc_{0}}{g_{0}},$$

$$\tilde{H}_{gb}=\frac{H_{gb}}{g_{0}}, \tilde{d}_{gb}=d_{gb}T, \tilde{D}_{gv}=\frac{D_{gv}T}{L^{2}}, \tilde{G}_{gvb}=\frac{G_{gvb}Tc_{0}}{g_{0}m_{0}^{2}}, \tilde{H}_{gv}=\frac{H_{gv}}{g_{0}}, \tilde{K}_{n}=\frac{K_{n}}{n_{0}}, \tilde{G}_{gvc}=\frac{G_{gvc}Tc_{0}}{g_{0}m_{0}^{2}},$$

$$\tilde{d}_{gv}=d_{gv}T, \tilde{D}_{n}=\frac{D_{n}T}{L^{2}}, \tilde{G}_{n}=\frac{G_{n}Tc_{0}}{n_{0}}, \tilde{d}_{n}=d_{n}T, \tilde{H}_{n}=\frac{H_{n}}{g_{0}}.$$

This resulted in the following set of **non-dimensional parameter values**:

$\tilde{D}_{hm} = 0.014$, $\tilde{K}_{hm} = 0.25$, $\tilde{C}_{kCTm} = 0.04$, $\tilde{K}_{kCTm} = 0.1$, $\tilde{C}_{kHTm} = 0.0034$, $\tilde{K}_{kHTm} = 0.5$, $\tilde{A}_{m0} = 1.01$, $\tilde{K}_{m} = 0.1$, $\tilde{\alpha}_{m} = 1$, $\tilde{Y}_{11} = 10$, $\tilde{H}_{11} = 0.1$, $\tilde{Y}_{12} = 2$, $\tilde{H}_{12} = 0.1$, $\tilde{Y}_{2} = 50$, $\tilde{H}_{2} = 0.1$, $\tilde{F}_{4} = 0.01$, $\tilde{D}_{f} = 0.02$, $\tilde{C}_{kf} = 0.4$, $\tilde{K}_{kf} = 0.1$, $\tilde{A}_{f0} = 0.202$, $\tilde{K}_{f} = 0.1$, $\tilde{\alpha}_{f} = 1$,$\tilde{d}_{f} = 0.1$, $\tilde{B}_{v} = 0.037$, $\tilde{B}_{ec} = 1.5$, $\tilde{Y}_{3} = 1000$, $\tilde{H}_{3} = 0.1$, $\tilde{A}_{c0} = 0.101$, $\tilde{K}_{c} = 0.1$, $\tilde{\alpha}_{c} = 1$, $\tilde{C}_{kb} = 1.4\cdot10^{-8}$, $\tilde{K}_{kb} = 0.01$ $\tilde{A}_{b0} = 0.202$, $\tilde{K}_{b} = 0.1$, $\tilde{\alpha}_{b} = 1$, $\tilde{d}_{b} = 0.1$, $\tilde{P}_{fs} = 0.2$, $\tilde{\kappa}_{f} = 1$, $\tilde{Q}_{f} = 1.5$, $\tilde{P}_{cs} = 0.2$, $\tilde{\kappa}_{c} = 1$, $\tilde{Q}_{c} = 1.5$, $\tilde{P}_{bs} = 2$, $\tilde{\kappa}_{b} = 1$, $\tilde{D}_{gc} = 0.005$, $\tilde{G}_{gc} = 50$, $\tilde{H}_{gc} = 1$, $\tilde{K}_{gc} = 0.1$, $\tilde{d}_{gc} = 100$, $\tilde{D}_{gb} = 0.005$, $\tilde{G}_{gb} = 500$, $\tilde{H}_{gb} = 1$, $\tilde{d}_{gb} = 100$, $\tilde{D}_{gv} = 0.5$, $\tilde{G}_{gvb} = 0.005$, $\tilde{H}_{gv} = 5$, $\tilde{K}_{n} = 0.05$, $\tilde{G}_{gvc} = 5.10^{5}$, $\tilde{d}_{gvc} = 1500$, $\tilde{D}_{n} = 0.014$, $\tilde{G}_{n} = 1$,$\tilde{d}_{n} = 18$,$\tilde{H}_{n} = 0.05.$

The system of equations must be complemented by suitable **initial and boundary conditions** to ensure the existence, uniqueness and non-negativity of a solution. At the start of the simulation, the entire callus area was filled with a loose fibrous tissue matrix (
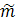
 = 0.1). All other variables were assumed to be zero initially. The mathematical model was closed by prescribing suitable boundary conditions. No-flux boundary conditions were applied for all variables carrying diffusion or taxis terms in their equations, except for the situations described below, where Dirichlet boundary conditions (i.e. concentration assigned at the boundary mimicking presence of that variable outside of the simulation domain) were prescribed for certain components on specific parts of the boundary and for a specified period of time.

Mesenchymal stem cells and fibroblasts were released into the callus tissue at the beginning of the healing process from three possible sources: the periosteum, the surrounding soft tissues and the marrow space at the site of the damaged cortical tissue [14]. All sources were adopted here (
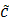
m_bc = 0.02 &
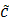
f_bc = 0.02 during first 3 days). The tip cells are initialized at specific starting positions, reflecting the intact vasculature in the cortical bone and marrow cavity [14]. Chondrogenic and osteogenic growth factors were assumed to originate from respectively the fractured bone ends and the cortex away from the fracture site. (
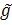
c_bc =
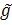
b_bc = 20 during respectively 5 and 10 days) [15,16].

***Implementation***

The 12 variables are non-negative. This qualitative property of the solution must be inherited by its numerically computed approximation because, amongst others, erroneous negative values for the concentrations might render otherwise stable reaction terms unstable. Besides ensuring non-negativity, the algorithm employed for the numerical solution of the model must respect conservation of mass. The finite volume technique was employed for its inherent mass conservation properties. The Method of Lines (MOL) was applied to separate the spatial and temporal discretisation. The axi-symmetric structure of the problem was employed to reduce the model to an equivalent problem in 2D space, leading subsequently to an efficient spatial discretisation.

The spatial domain was covered with an equidistant computational grid. After convergence tests the grid size was fixed at 0.05 mm in both directions. On this grid, the diffusion and reaction terms in the system of equations (1-11) were discretized using respectively the standard second order central difference approximation and pointwise evaluation, which were found to be sufficient in terms of accuracy and to ensure non-negativity of the solution of the resulting ordinary differential equation (ODE) system [17]. Contrarily, the discretisation of the taxis terms in this system of equations required the application of upwinding techniques with nonlinear limiter functions (van Leer limiter) to guarantee accurate, non-negative solutions of the MOL-ODE system. The order of the spatial approximation is two in general. For the time integration of the resulting stiff MOL-ODE system the code ROWMAP [18] was used. The methods built-in automatic step size control ensures the error caused in each time step (local error) to remain below a user-prescribed tolerance while keeping the computational cost as low as possible.

For a discussion of the simplifications related to the methods described above, we refer the reader to Geris et al. and Peiffer et al. [13,19].

**Reference List**

1. Pountos I, Giannoudis PV (2005) Biology of mesenchymal stem cells. Injury 36: 8-12.

2. Fiedler J, Leucht F, Waltenberger J, Dehio C, Brenner RE (2005) VEGF-A and PlGF-1 stimulate chemotactic migration of human mesenchymal progenitor cells. Biochem Biophys Res Commun 334: 561-568.

3. Olsen L, Sherratt JA, Maini PK, Arnold F (1997) A mathematical model for the capillary endothelial cell-extracellular matrix interactions in wound-healing angiogenesis. IMA J Math Appl Med Biol 14: 261-281.

4. Sherratt JA (1994) Chemotaxis and Chemokinesis in Eukaryotic Cells - the Keller-Segel Equations As An Approximation to A Detailed Model. Bull Math Biol 56: 129-146.

5. Weinberg CB, Bell E (1985) Regulation of Proliferation of Bovine Aortic Endothelial-Cells, Smooth-Muscle Cells, and Adventitial Fibroblasts in Collagen Lattices. J Cell Physiol 122: 410-414.

6. Street J, Bao M, deGuzman L, Bunting S, Peale FV et al. (2002) Vascular endothelial growth factor stimulates bone repair by promoting angiogenesis and bone turnover. PNAS 99: 9656-9661.

7. Bailon-Plaza A, van der Meulen MC (2001) A mathematical framework to study the effects of growth factor influences on fracture healing. J Theor Biol 212: 191-209. 10.1006/jtbi.2001.2372 [doi];S0022-5193(01)92372-0 [pii].

8. Rossi F, MacLean HE, Yuan W, Francis RO, Semenova E et al. (2002) p107 and p130 coordinately regulate proliferation, Cbfa1 expression, and hypertrophic differentiation during endochondral bone development. Dev Biol 247: 271-285.

9. Einhorn TA (1998) The cell and molecular biology of fracture healing. Clin Orthop Rel Res S7-S21.

10. Harrison LJ, Cunningham JL, Stromberg L, Goodship AE (2003) Controlled induction of a pseudarthrosis: A study using a rodent model. J Orthop Trauma 17: 11-21.

11. Nogami H, Oohira A (1984) Postnatal New Bone-Formation. Clin Orthop Rel Res 106-113.

12. Joyce ME, Terek RM, Jingushi S, Bolander ME (1990) Role of Transforming Growth-Factor-Beta in Fracture Repair. Ann NY Acad Sci 593: 107-123.

13. Geris L, Gerisch A, Sloten JV, Weiner R, Oosterwyck HV (2008) Angiogenesis in bone fracture healing: a bioregulatory model. J Theor Biol 251: 137-158. S0022-5193(07)00567-X [pii];10.1016/j.jtbi.2007.11.008 [doi].

14. Gerstenfeld LC, Cullinane DM, Barnes GL, Graves DT, Einhorn TA (2003) Fracture healing as a post-natal developmental process: Molecular, spatial, and temporal aspects of its regulation. J Cell Biochem 88: 873-884.

15. Barnes GL, Kostenuik PJ, Gerstenfeld LC, Einhorn TA (1999) Growth factor regulation of fracture repair. J Bone Miner Res 14: 1805-1815.

16. Dimitriou R, Tsiridis E, Giannoudis PV (2005) Current concepts of molecular aspects of bone healing. Injury 36: 1392-1404.

17. Gerisch A, Chaplain MAJ (2006) Robust numerical methods for taxis-diffusion-reaction systems: Applications to biomedical problems. Math Comput Model 43: 49-75.

18. Weiner R, Schmitt BA, Podhaisky H (1997) ROWMAP - a ROW-code with Krylov techniques for large stiff ODEs. Appl Numer Math 25: 303-319.

19. Peiffer V, Gerisch A, Vandepitte D, Van OH, Geris L (2011) A hybrid bioregulatory model of angiogenesis during bone fracture healing. Biomech Model Mechanobiol 10: 383-395. 10.1007/s10237-010-0241-7 [doi].
